# Paleohistology of *Caraguatypotherium munozi* (Mammalia, Notoungulata, Mesotheriidae) from the early late Miocene of northern Chile: A preliminary ontogenetic approach

**DOI:** 10.1371/journal.pone.0273127

**Published:** 2023-03-16

**Authors:** Jorge Campos-Medina, Germán Montoya-Sanhueza, Karen Moreno, Enrique Bostelmann Torrealba, Marcelo García

**Affiliations:** 1 Instituto de Ciencias de la Tierra, Facultad de Ciencias, Universidad Austral de Chile, Valdivia, Región de Los Ríos, Chile; 2 Centro de Estudios Avanzados en Zonas Áridas (CEAZA), Coquimbo, Chile; 3 THERIUM SPA, Paleontología y Patrimonio, Curicó, Región del Maule; 4 Department of Zoology, Faculty of Science, University of South Bohemia, České Budějovice, Czech Republic; 5 Doctorado en Ciencias Mención Ecología y Evolución, Universidad Austral de Chile, Valdivia, Región de Los Ríos, Chile; 6 Museo Regional de Aysén, Coyhaique, Región de Aysén; 7 Departamento de Geología, Universidad de Chile, Santiago, Chile; Laboratoire de Biologie du Développement de Villefranche-sur-Mer, FRANCE

## Abstract

The Miocene Caragua fossil fauna in northern Chile contains a considerable number (7) of articulated partial skeletons tentatively assigned to *Caraguatypotherium munozi* (Notoungulata, Mesotheriidae), which presents up to 40% body size difference. Since either inter- and intra- specific wide size range has been observed in the Mesotheriidae family in general, we wanted explore the ontogenic stage signature of the sample, by carrying out the first comprehensive paleohistological description of the appendicular system in Notoungulata. Results show that: 1) they can be classified as subadults and adults, based on the presence of bone tissues typical of ceased somatic growth; 2) there is a notorious inter-skeletal variation on bone growth rates (skeletal modularity), particularly, the humerus showed a slower diameter growth and less remodelling than the femur, resulting as a better element for ontogenetic analyses; 3) marked cyclical growth is observed, characterised by fast early ontogenic continuous growth, and subsequent fast/slow stratified bone tissue layering. In general, such growth pattern suggests that *C*. *munozi* had a similar ontogenetic growth process as other modern mammals, that it should also be influenced by other sex-related, ecological and environmental factors. Likely related to the presence of rapid climatic variations, due to orogenic uplift and concomitant re-organization of the drainage processes along the western tectonic front of the Central Andes at that time.

## Introduction

During the Cenozoic, the South American native ungulates (SANUs) represented a particular and diverse group of phylogenetically related clades, presently grouped in the orders Xenungulata, Pyrotheria, Astrapotheria, Notoungulata and Litopterna, plus few endemic, early Paleogene “condylarthra” [1–3]. These extinct mammals exhibited one of the most divergent variety of body sizes and morphological adaptations, including fully cursorial locomotion in the medium sized Proterotheriidae [4], mass graviportal standing in gigantic toxodontids, astrapotheres, and pyrotheres [5–8], and scansorial, cursorial and semi-fossorial habits in diverse lineages within the small to medium-sized typotheres [9–11]. Studies based on collagen protein chains (proteomics) extracted from Pleistocene fossils (i.e., Toxodontia and Litopterna), have placed SANUs within Laurasiatheria, specifically as a sister group to extant Perissodactyla (e.g. tapirs, horses and rhinoceroses) [12–14]. Nevertheless, recent morphological phylogenetic studies have proposed alternative relationships, identifying them as a non-monophyletic group [4, 8, 15, 16]. In this sense the study of their biology and evolution have represented one of the most challenging tasks for vertebrate palaeontologists since the end of the 19th century [1, 15]. Although some aspects of their paleobiology such as their locomotor modes and general lifestyle have been documented (e.g. [9, 16–18]), other important aspects such as their skeletal growth and development remain largely unknown (but see [19] for cranial features).

Paleohistology is a useful tool for elucidating the growth patterns, life history and physiology of extinct vertebrates [20–25]. It has provided insights on several aspects of the evolutionary biology of extinct mammals including the reconstruction of their growth and development [22, 26–29], adaptations to different lifestyles [27], ecology [22, 29, 30] and physiology [22, 27]. However, the studies on the paleohistology of extinct mammals of South American faunas are still scarce. For example, de Ricqlès *et al*. [31] reviewed the paleohistological collection of Paul Gervais in Paris (Laboratory of Comparative Anatomy of the Museum of Natural History), and briefly described some elements of notoungulates such as an ulna of *Toxodon* sp. (Toxodontidae), and various cross-sections of ulna, molars and incisors initially assigned to Typotherium, and presently recognized as *Mesotherium* sp. [32, 33] (Mesotheriidae, Simpson, 1940). More recently, *Tomassini et al*. [34, 35] analysed the paleohistology of *Paedotherium bonaerense* (Hegetotheriidae) and *Toxodon platensis*, and documented fossil-diagenetic modifications in their bone microstructure. Forasiepi *et al*. [36] and Carrillo and Asher [37] also provided a general paleohistological description of a new toxodontid species (*Nesodon taweretus*), and of an extremely well-preserved skeleton of *Thomashuxleya externa* (Isotemnidae) respectively. Houssaye *et al*. [18] analysed the bone microanatomy of several SANUs including Pyrotheria, Astrapotheria and Notoungulata. The latter authors quantified the relative proportions of compact and trabecular bone regions using CT scans to infer locomotor adaptations, although no paleohistological descriptions were included.

Considering this information, no attempts to assess the growth patterns and development of these extinct mammals throughout bone paleohistology have been done. Clearly, the study of ontogenetic series including several individuals, more taxonomically restricted taxa, as well as the incorporation of different bone elements, would largely improve our knowledge on the paleobiology of these extinct animals.

Mesotheriids and specifically the subfamily Mesotheriinae are one of the most late diverging groups within the suborder Typotheria, being common faunal elements during the Neogene at mid-latitude Andean localities, and lowland Pampean ecosystems (for a detailed account of their diversity, evolution, taxonomy and distribution see [38–45], until their final disappearance during the middle Pleistocene (Chibanian) [44, 45]. Despite this, many aspects of their biology and paleoecology are largely unknown. Particularly interesting is the presence of large cranio-dental size differences within ontogenetic series in various Mesotheriidae [33, 40, 46].

The present study describes in detail the long bone paleohistology of several individuals assigned to *C*. *munozi*, based on their restricted stratigraphical and local distribution at the Miocene Copaquilla basin, northern Chile. Because the material collected exhibits clear differences in body size among individuals (Table 1) it becomes fundamental to uncover the real nature of such variation, whether resulting from ontogenetic differences or the presence of multiple taxa in the region. Based on this observation, we hypothesise that the smaller individuals represent young individuals of *C*. *munozi*, and therefore their bones will exhibit typical osteohistological features of mammalian juveniles/subadults, such as disorganised and relatively fast deposition of bone tissues, highly vascularized cortices and comparatively lower levels of secondary bone remodelling. In contrast, larger individuals will reflect more advanced ontogenetic stages, with typical osteohistological features of adult mammals, such as slowly deposited bone tissues, increased bone remodelling, and evidence of growth marks. This study discusses the potential factors explaining the morphological variation of the remains assigned to *C*. *munozi*, in terms of ontogeny, intraspecific variation, and interskeletal variation, thus providing a basis for future palaeohistological comparisons of Mesotheriidae and other Notoungulates.

**Table 1:** Bone measurements (cm) of seven individuals of *Caraguatypotherium munozi* from Huaylas formation.

INDIVIDUALS and code	Bone element	Total length	Inferred midshaft length (craniodistal, antero/posterior)	Anteroposterior diameter at the midshaft (approx. 50% of total length)	Lateromedial diameter at the midshaft (approx. 50% of total length)
Individual 1: HUAY15-100					
GEOUACH.HS.HI.1	Left humerus	20.5	10.23	2.75	2.29
GEOUACH.HS.MTC.2	Left Mc III	7.24	3.62	0.85	1
GEOUACH.HS.PFI.1	1^st^ left phalange	2.79	1.395	0.62	1.02
Individual 2: HUAY15-200					
GEOUACH.HS.HD.1	Right humerus*	19.53	9.5	2.74	1.57
GEOUACH.HS.UD.1	Right ulna**	23.2	11.6	2.32	1.1
GEOUACH.HS.PFD.1	1^st^ right phalange	2.55	1.2	0.64	0.84
GEOUACH.HS.FI.1	Left femur***	20.44	10.5	1.4	2.25
Individual 3: HUAY17-05					
GEOUACH.HI.2	Left humerus*	12.4	6.2	1.64	1.24
Individual 4: HUAY16-100					
GEOUACH.HS.TI.1	Left tibia*	13.3	6.65	1.55	1.09
GEOUACH.HS. MTSI.1	Left Mt II	4.48	2.42	0.57	0.81
Individual 5: HUAY15-084					
GEOUACH.HS.FI.2	Left femur***	16.4	8.2	1.46	1.81
GEOUACH.HS.TI.2	Left tibia*	12.41	6.2	1.4	1.04
Individual 6: HUAY15-027					
GEOUACH.HS.TI.3	Left tibia***	17.4	8.7	1.7	1.21
Individual 7: HUAY17-02					
GEOUACH.HS.FD.1	Right femur**	23.3	11.65	11	-

Total bone length and corresponding midshaft was inferred in the material were the:

*proximal epiphysis is missing,

**distal epiphysis is missing; or

***both epiphyses are missing

### Geological context

**Fig 1:**
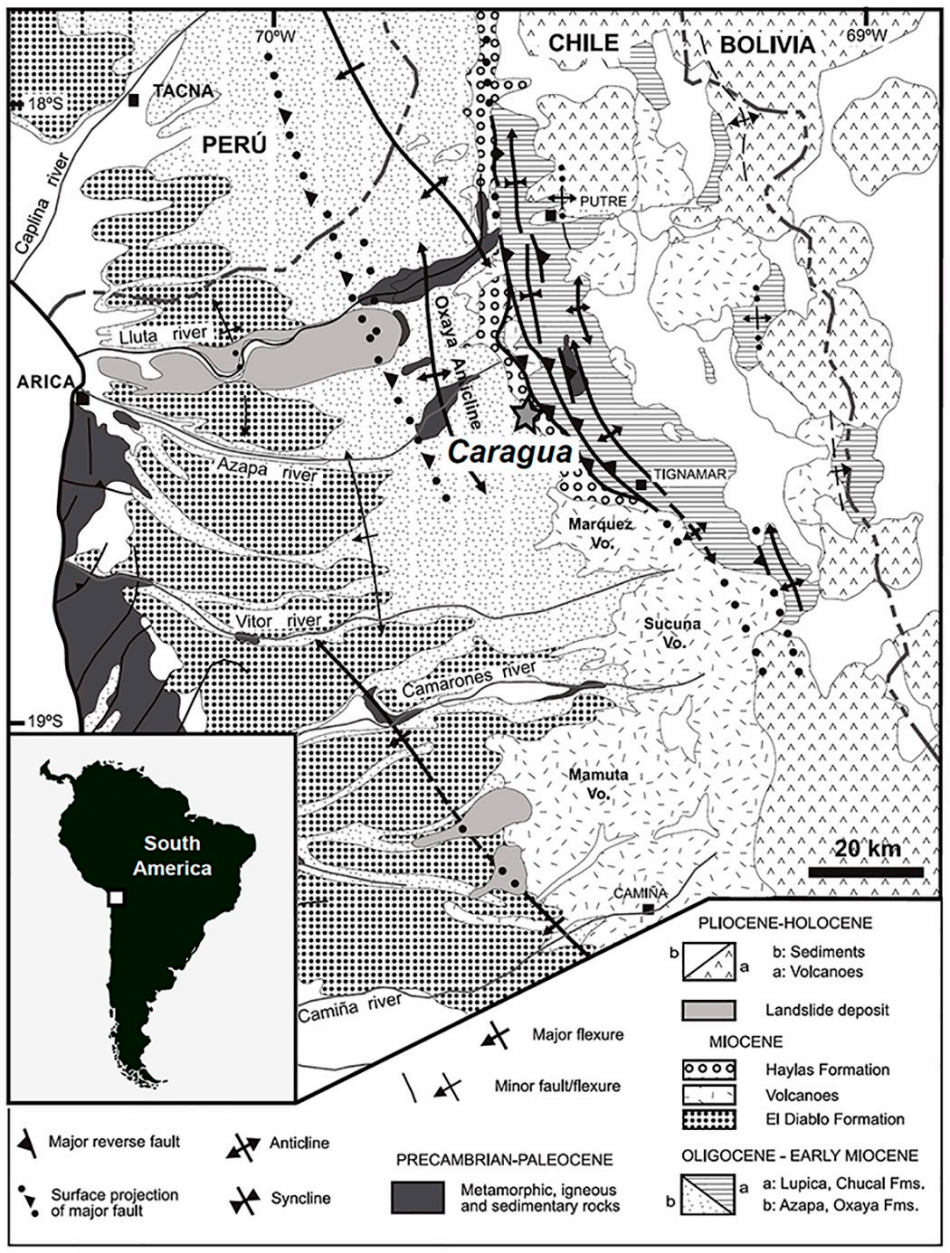
Geographic and geological map. Geographic and geological setting of the Caragua fossil locality (star), in northern Chile, showing the main lito-stratigraphic units and structural features; adapted from García et al. [48].

The Caragua vertebrate assemblage was collected from the Huaylas Formation [47–49], exposed in the eastern Precordillera of Arica at ~18.5°S, currently at 2.900–3100 m.a.s.l. (Fig 1). To the west of Caragua, the Precordillera consists of the late Miocene Oxaya Anticline, which is a large, west-vergent, gentle fold; to the east, the Western Cordillera is formed by a Miocene fold-and-fault belt involving the Precambrian-Paleozoic metamorphic basement [47, 49–51]. The Huaylas Formation consists of polymictic sandstones and semi-consolidate gravels, which were deposited predominantly in a fluvial environment and subordinately in an alluvial setting. K-Ar and Ar/Ar dates of volcanic rocks indicate that the fossiliferous beds were deposited between 11.7 ± 0.7 Ma and 10.7 ± 0.3 Ma, early late Miocene [47–49] (Tortonian, [52]).

The fluvial-alluvial environment of the Huaylas Formation rocks and the presence of middle Miocene episodic drainage systems reflecting a semi-arid climate contrasts with the modern hyper-arid climate conditions present in the region [48, 53, 89]. This changing environmental setting represents a unique opportunity to assess the role of major geomorphological processes on the associated fauna.

## Materials and methods

### Material of *Caraguatypotherium munozi*

The Caragua fossil fauna include several mammals [54–58], in which *C*. *munozi* is the most abundant [58] and represented by a diversity of cranial and postcranial elements with variable degrees of preservation. Multiple appendicular elements of seven individuals were analysed. Taxonomical assignation to *C*. *munozi* was based on anatomical similarities and proportions, dental homologies, and their restricted stratigraphical and localised spatial distribution in the outcrops. Three individuals include cranial and dental elements (HUAY15-100; HUAY15-200; HUAY17-05). The fossil materials were collected during a series of field campaigns in the Huaylas Formation between 2015 and 2017 (List of materials: Table 1). The materials are currently deposited at the Instituto de Ciencias de la Tierra, Universidad Austral de Chile.

### Postcraneal bone morphology and external measurements

The bone material was measured either directly with a digital calliper or using a software of image processing, ImageJ [59], after reconstruction. Because several bones were fragmented or lacked one or both epiphyses, full reconstructions of the bone material was based on the comparison with more complete skeletons of closely related mesotheriines (e.g. *Plesiotypotherium* [58, 60, 61]). This allowed us to ensure that our histological samples are come from the midshaft. Three linear measurements of the long bones were obtained: the total length of the bone, measured from the proximal articular surface to the distal articular surface; and the anteroposterior and mediolateral diameters of the diaphysis, both taken approximately at the midshaft (around 50% from the proximal articular surface) (Table 1). We also assessed the gross skeletal maturity of each element (when possible) by classifying the degree of epiphyseal fusion (adult stage), as fused and not fused (juvenile stage) (Table 1) given the highly conservative shape of the appendicular skeleton in mesotherids [9, 61]. Virtual completion of bone elements from scaled images was performed using Adobe Photoshop CC and Adobe Illustrator CC (Adobe Systems Inc.).

High quality photographs of each bone in anterior, posterior, medial, and lateral views were taken using a DSLR camera Canon EOS Rebel T6. Digital replicas of the fossils were created with a Go!SCAN 3D.20 scanner using the VXElements software (Creaform Inc.). All procedures were carried out at the Laboratorio de Observación y Documentación, at the Instituto de Ciencias de la Tierra (Universidad Austral de Chile, Valdivia). Additionally, silicone replicas were created following the methodology used by Reuil and Muzzopappa [62], at the Laboratorio de Paleontología (Universidad Austral de Chile, LabPALEO-UACH).

### Paleohistological procedures

A total of fourteen paleohistological cross-sections were obtained from forelimbs and hindlimbs of seven individuals (Table 1). Thin sections were prepared following standard histological protocols defined by Chinsamy and Raath [63]. The preparation and housing of specimens corresponds to LabPALEO-UACH. Cross-sections were performed at the midshaft of the diaphysis, i.e., ~50% from the proximal articular surface. This region of the bone is usually not excessively remodelled or resorbed in mammals as compared to the more proximal and distal regions of the bone, thus preserving a relatively complete sequence of the animal growth (i.e. [21, 27, 30, 64–68]).

**Fig 2:**
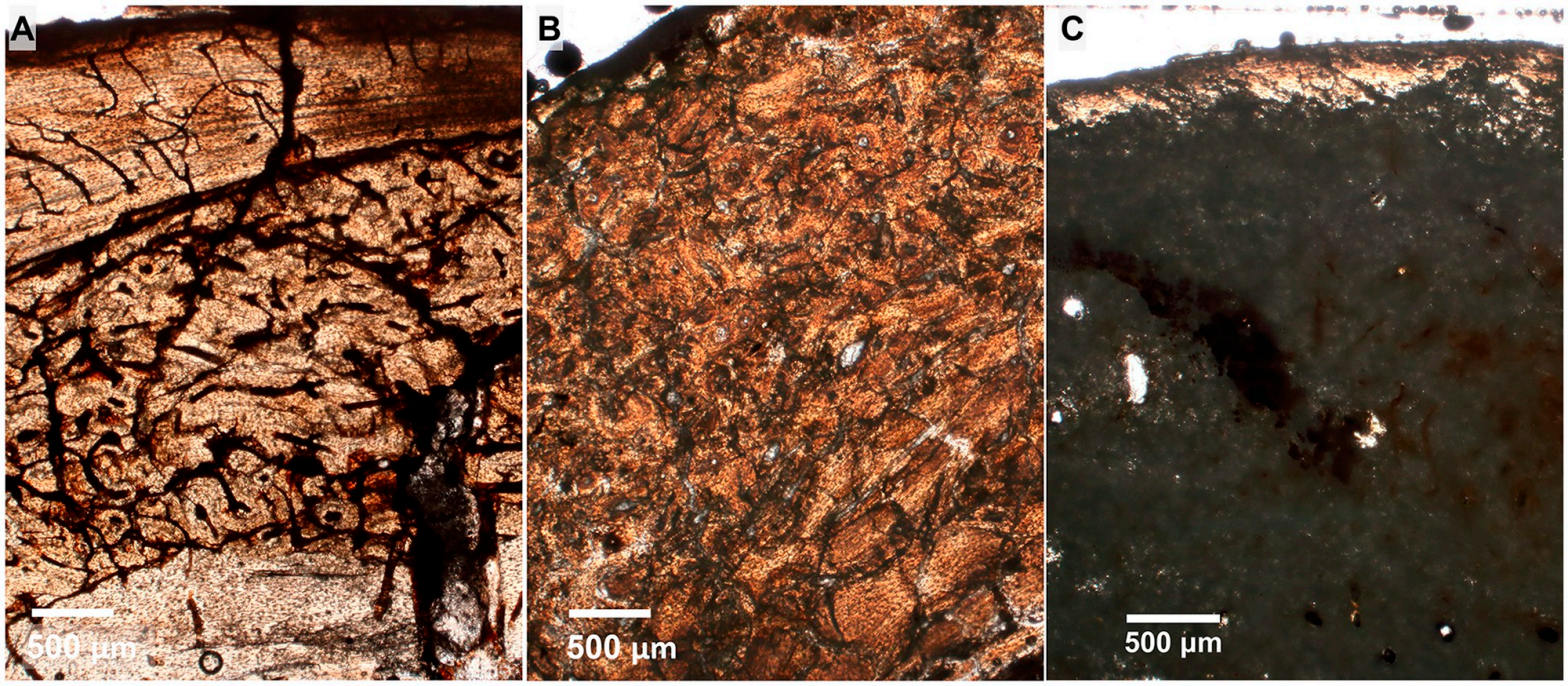
Degree of preservation in specimens of *Caraguatypotherium munozi* following the methodology used by Hollund et al. [69]. (A) State 1, well preserved (GEOUACH.HS.HD.1); (B) State 2, intermediate preservation (GEOUACH.HS.FD.1); (C) State 3, poor preservation (GEOUACH.HS.FI.1).

Because of the varying degrees of preservation of the bone material, we qualitatively classified them into three categories based on the degree of histological preservation [69]. These categories are based on the capacity to observe histological structures under the petrographic microscope (Fig 2): State 1 (well preserved), characterised by a relatively complete integrity of the bone microstructure; State 2 (intermediate preservation), refers to the partial preservation of histological characteristics in the section; and State 3 (poor preservation), with null or very scant observation of histological features in the section.

### Paleohistological nomenclature

**Fig 3:**
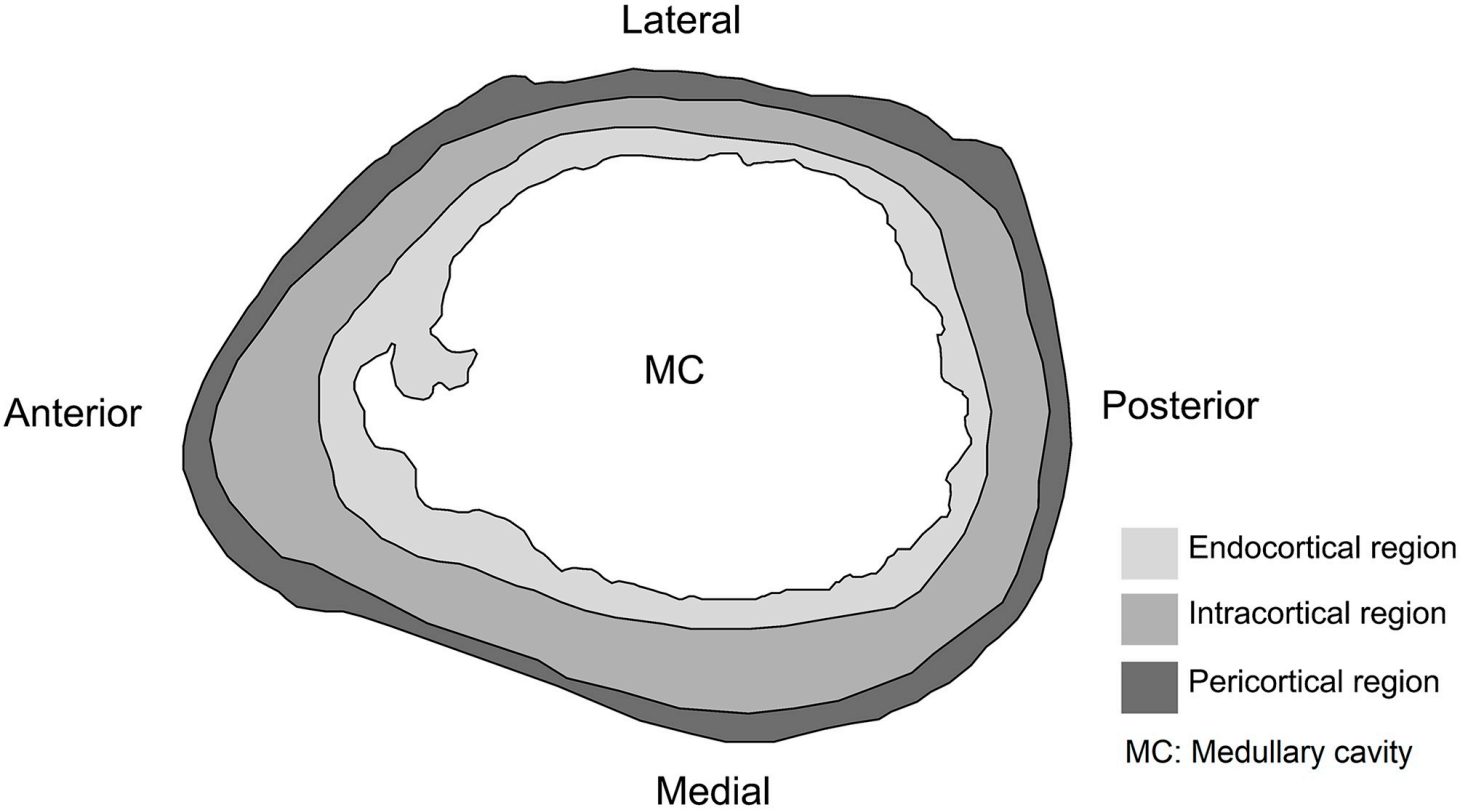
Right humerus cross-section diagram. Divisions of bone cortex used to describe paleohistological samples in *Caraguatypotherium munozi*.

A detailed qualitative description of the anterior, posterior, lateral and medial sides of the cross-section was carried out for each bone element. For this, the bone cortex was arbitrarily divided into three regions: endocortical, intracortical and pericortical (Fig 3) [68].

The description of the bone microstructure was carried out using an optic petrographic microscope Zeiss Axio Scope A1 and polarised light with a lambda filter to observe the direction of collagen fibres. Histological descriptions of the bone tissues were made following the classical studies on vertebrate osteohistology (Enlow [70], Francillon-Vieillot *et al*. [71] and de Ricqlès *et al*. [72]). We identified the shape and relative size of the osteocyte lacunae, the orientation of the mineralized collagen fibres, and the predominant matrix type of the cortex and its vascularization.

#### Types of bone matrix according distinct ontogenetical stages

1. Woven (WB) and fibrolamellar bone (FLB): composed of collagen fibres of multidirectional cortical distribution, monorefringent under the microscope with polarised light (independent of the longitudinal or transverse direction of the cut). Typically, this type of bone tissue presents randomly distributed and globular osteocyte lacunae and is usually associated with a high rate of osteogenesis and high vascularization. If primary osteons are present within the WB, having a more anisotropic structure, it is termed as FLB.
2. Compacted coarse cancellous bone (CCCB): type of bone found primarily in the epiphyseal plates and that through modelling can be encountered in the middle part of the diaphysis [68]. In young individuals, the ends of their long bones comprise endochondral cancellous bone, but as the animal matures the "ends" become incorporated into the bone wall of the older individual. In the process, the cancellous bone becomes compacted with lamellar bone deposits which fill in the cancellous spaces. The bone tissue characteristically has a swirled, convoluted appearance.
3. Parallel-fibered bone (PFB): Composed of tightly packed collagen fibres, usually with the same orientation, mono or birefringent under polarised light microscope (depending on the transverse or longitudinal direction of the cut). It presents flattened and relatively organised osteocyte lacunae. This type of bone is usually associated with a lower osteogenesis rate as compared with WB.
4. Lamellar bone (LB): It presents the higher degree of organisation of collagen fibres and is composed of successive thin layers called lamellae that correspond to tightly packed collagen fibres. The visualisation under polarised light microscope is variable (mono or birefringent). Each lamella usually contains rows of flattened osteocyte lacunae. This type of bone tissue is usually associated with a lower rate of osteogenesis and poor vascularization as compared to PFB.

#### Ontogenetic paleohistology

In general, the postnatal morphogenesis of long bones of mammals follows a relatively conserved pattern of bone matrix formation, from more rapidly deposited bone tissues (e.g., WB) to slowly deposited bone tissues (e.g., LB) (see [65]), and references therein). This generalised sequence of bone tissue formation has been useful to identify different ontogenetic stages in extinct mammalian species [21, 73]. Thus, predominantly more disorganised bone matrices with large and globular osteocyte lacunae and lacking any obvious organisation of their collagen fibres (e.g. WB) would account for a relatively less mature bone and therefore to earlier stages of development (e.g. juvenile individuals). In contrast, a more mature bone would display a higher degree of organisation of its collagen fibres and bone matrix, with usually smaller osteocyte lacunae of fusiform shape (e.g., LB), which is typical of more advanced stages of somatic maturity (i.e. adult individuals).

It has been reported that the cease of somatic growth in some vertebrates is characterised by the formation of a layer of periosteal lamellar bone (PLB), generally called external fundamental system (EFS) by some authors [27] or outer circumferential layer (OCL) by others [70]. This is a clear indicator of low rates of bone deposition, characteristic of many vertebrates including mammals in advanced stages of somatic maturity (e.g. [21, 27, 68]).

Overall, to describe bone growth, we make emphasis on describing the processes of bone modelling finally observable at the midshaft, which involves the formation and resorption of bone tissues in a coupled or uncoupled mechanism acting in different bone surfaces [68]. This process is responsible for the main longitudinal and diametrical growth of the bone, thus resulting in both shape and size changes during ontogeny [68]. Bone modelling is synonymous to Enlow’s [70] term “growth remodelling”. We also describe bone remodelling, which refers to the formation of Haversian systems, sometimes resulting in dense aggregations of secondary osteons covering the bone surface, termed dense Haversian bone [70].

It is important to consider that the above definitions are not definitive, mainly because the bone matrix composition of a single element and/or one individual can vary considerably in its arrangement (e.g. [64, 65, 74, 75]). For this reason, to properly determine ontogenetic stages in individuals, it is necessary to consider the assessment of complete transversal sections of different bone, and when possible, include multiple elements to assess inter-skeletal variation. Other histological features associated with ontogeny in mammals include the development of secondary osteons and its extension over the bone surface (bone remodelling and development of Haversian bone). Considering that each individual of *C*. *munozi* possess more than one bone element, we summarize the ontogenetic inferences obtained from the paleohistological description for each individual. The final ontogenetic stage is defined by the elements exhibiting the most active bone modelling activity, and are described from the most proximal to the most distal elements.

## Results

**Fig 4:**
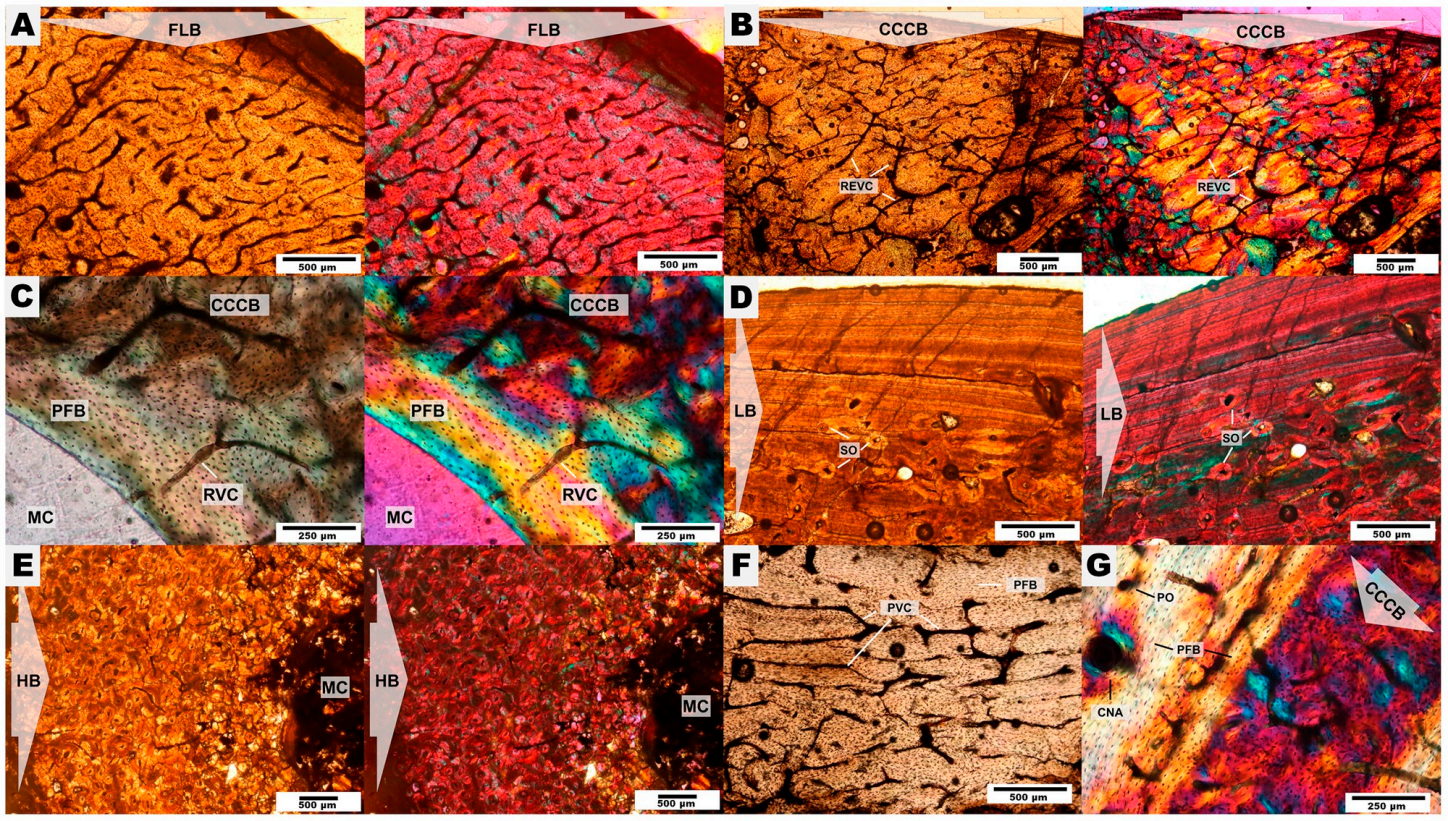
Bone tissue types observed in *Caraguatypotherium munozi*. (A) Fibrolamellar bone (FLB);(GEOUACH.HS.UD.1); (B) Compacted coarse cancellous bone (CCCB) and reticular vascular canals (REVC);(GEOUACH.HS.UD.1); (C) Parallel fibered bone (PFB), radial vascular canals (RVC) and compacted coarse cancellous bone (CCCB);(GEOUACH.HS.PFD.1); (D) Lamellar bone (LB) and secondary osteons (SO);(GEOUACH.HS.TI.2); (E) Haversian bone (HB);(GEOUACH.HS.FI.2); (F) Plexiform vascular canal (PVC) and parallel fibered bone (PFB);(GEOUACH.HS.UD.1); (G) Primary osteon (PO), Parallel fibered bone (PFB), Canal of the nutrient artery (CNA) and Compacted coarse cancellous bone (CCCB);(GEOUACH.HS.PFD.1).

Distinct bone tissue matrices were present in the appendicular skeleton of *C*. *munozi*: fibrolamellar bone (FLB), compacted coarse cancellous bone (CCCB) parallel fibered bone (PFB), lamellar bone (LB), and Haversian bone (HB). All the sections showed varying amounts of some of these tissues. Simple vascular canals (VC), radial vascular canals (RVC), reticular vascular canals (REVC), plexiform vascular canals (PVC), canals of the nutrient arteries (CNA), resorption cavities (RC), as well as primary (PO) and secondary osteons (SO), were also found in the sections (Fig 4). General descriptions of these bone tissue types and its distribution within the cross-sections is presented below.

### Histodiversity and bone growth

#### Humerus

**Fig 5:**
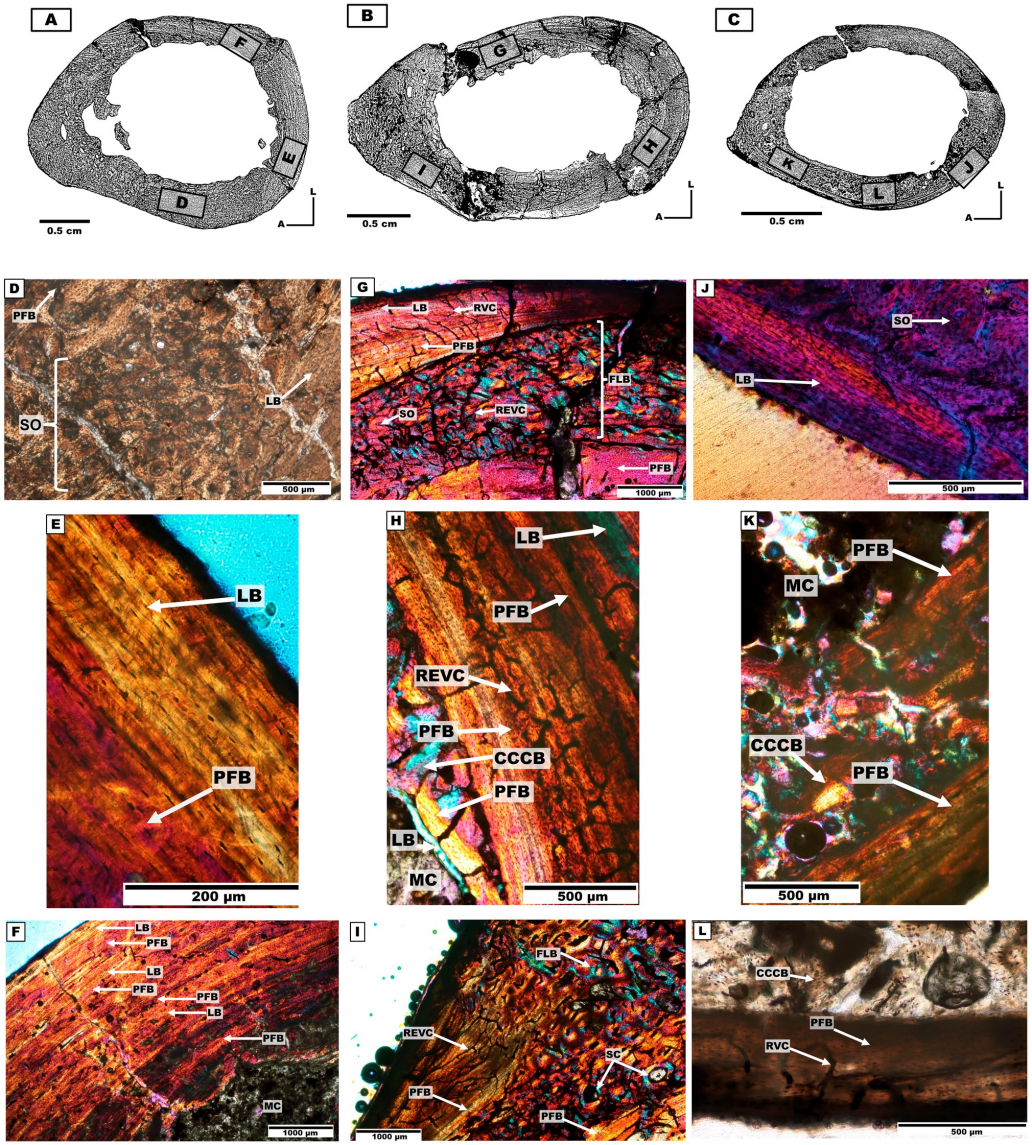
Distinct humerus cross-sections of the mesotheriine notoungulate *Caraguatypotherium munozi* from the middle Miocene of Caragua, Huaylas formation. (A) Humerus cross-section for individual 1 (GEOUACH.HS.HI.1); (B) Humerus cross-section for individual 2 (GEOUACH.HS.HD.1); (C) Humerus cross-section for individual 3 (GEOUACH.HI.2). (D) PFB in the endocortical region, SO in the intracortical region, LB in the pericortical region (GEOUACH.HS.HI.1); (E) PFB in the intracortical region and LB in the pericortical region (GEOUACH.HS.HI.1); (F) Intercalation of LB and PFB in the posterior area (GEOUACH.HS.HI.1); (G) Endocortical and pericortical region presence of LB and PFB, intracortical region presence FLB and SO (GEOUACH.HS.HD.1); (H) LB in the endocortical region, CCCB, PFB and LB in the intracortical region (GEOUACH.HS.HD.1); (I) Anterior area, FLB in the intracortical region, in the lateral area PFB and abundant vascularity (GEOUACH.HS.HD.1); (J) SO in the intracortical region, LB in the pericortical region (GEOUACH.HI.2); (K) PFB in the endocortical and pericortical region, CCCB in the intracortical region (GEOUACH.HI.2); (L) PFB in the pericortical region, RVC in this region open, in the intracortical region CCCB (GEOUACH.HI.2). For abbreviations refer to Fig 4.

*Bone microanatomy*. Three distinct humeri were analysed for distinct individuals: Individual 1, individual 2 and individual 3 (Table 1, Fig 5A–5C). The cross-section is ellipsoidal, elongated in antero-posterior direction, probably because of the development of both the deltopectoral crest in the anterior side and the supracondylar crest in the posterolateral side of the diaphysis [61]. The three individuals exhibited an open medullary cavity with fragmented remains of trabecular organisation, mostly localised in the anterior side.

*Bone microstructure*. The endocortical region is composed of bands of LB and PFB of different thicknesses that surround the medullary cavity (Fig 5D, 5G, 5H and 5K). The vascularization of the endocortical region is mostly of radial type.

The intracortical region is highly variable in bone tissue composition. The intracortical region of the humerus of individual 3 is almost completely composed of CCCB (Fig 5K), and contrasts with the other individuals in that they exhibit both CCCB and FLB in the anterior side (Fig 5I). The medial and lateral sides are composed of FLB, with development of SO in the individual 1 (Fig 5D and 5E), while the posterior region exhibited intercalations of PFB and LB, with presence of PO and SO (Fig 5F and 5H). Conspicuous vascularization is observed in the anterior side, corresponding to REVC and RVC (Fig 5I).

The pericortical region of the humeri exhibited both PFB and LB (Fig 5G, 5K and 5L). These tissues surround the whole bone section, and are thinner or absent in the anterior side, where CCCB and FLB predominate. The posterior side of the humeri presented relatively thicker bands of well-defined LB as compared to the rest of the cross-section (Fig 5F and 5H), and the three humeri of individuals 1–3 present EFS. The pericortical vascularization of the humerus of individuals 1 and 2 is minimal or absent in comparison to the humerus of individual 3, which exhibited mostly RVC and REVC, sometimes opened to the external surface (Fig 5L), thus indicating active osteogenesis.

#### Ulna

**Fig 6:**
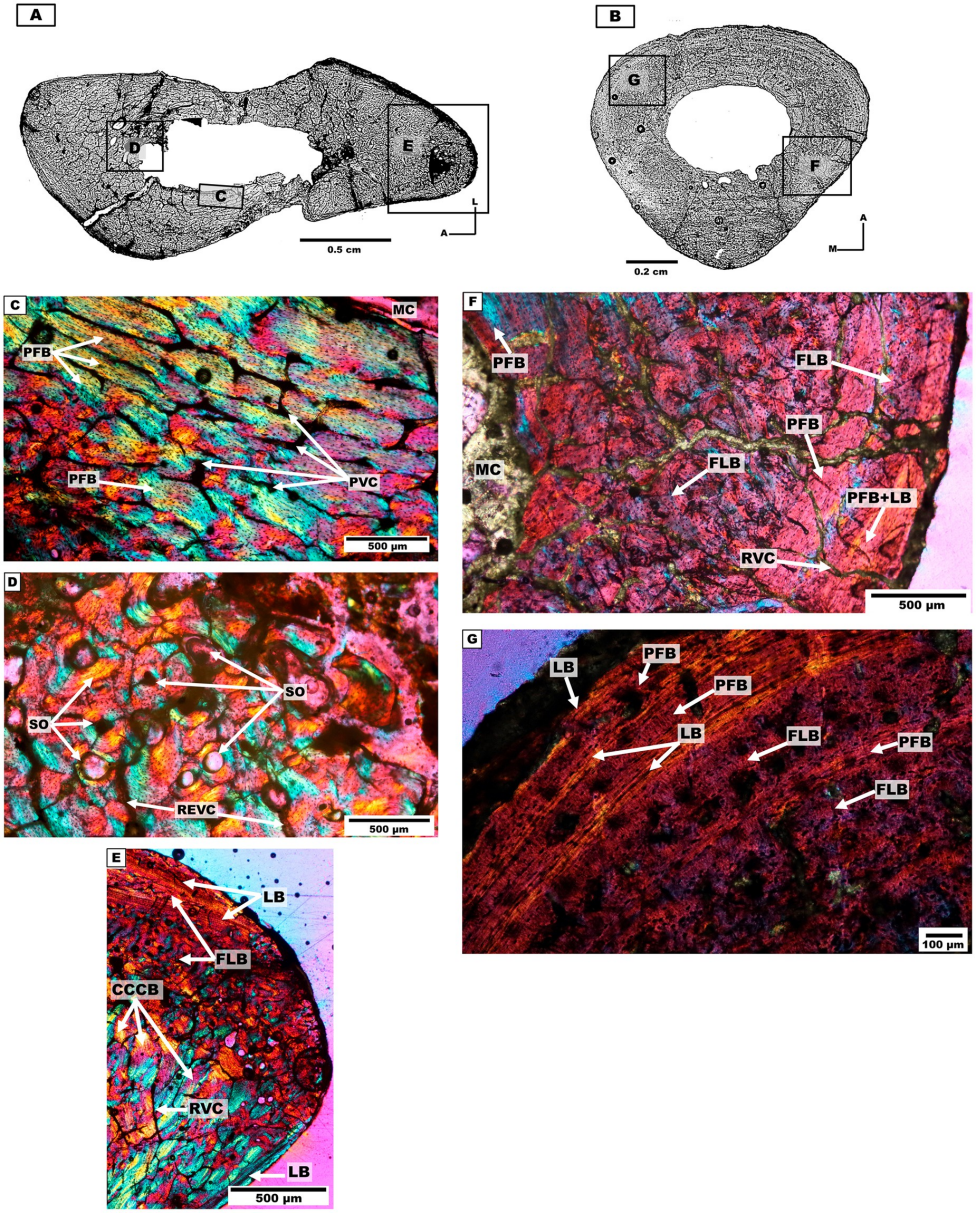
Ulnar and metacarpal III cross-sections of the mesotheriine notoungulate *Caraguatypotherium munozi* from middle Miocene of Caragua, Huaylas formation. (A) Ulna cross-section for individual 2 (GEOUACH.HS.UD.1); (B) Metacarpal III cross-section for individual 1 (GEOUACH.HS.MTC.2); (C) Intracortical region, presence of PFB with PVC (GEOUACH.HS.UD.1); (D) SO, in the endocortical region (GEOUACH.HS.UD.1); (E) Posterior area of the section, presence of CCCB, LB and PFB are identified. Vascularization dominated by RVC (GEOUACH.HS.UD.1); (F) PFB in the endocortical region, FLB in the intracortical region PFB and LB in the pericortical region GEOUACH.HS.MTC.2); (G) In the intracortical region and pericortical region intercalation of LB with PFB (GEOUACH.HS.MTC.2). For abbreviations refer to Fig 4.

*Bone microanatomy*. The ulna of the individual 2 was analysed (Table 1, Fig 6A). It is clearly elliptical, with the antero-posterior axis of the section being much longer than the medio-lateral axis. There is a conspicuous medullary cavity, also of ellipsoidal shape, with presence of trabecular bone.

*Bone microstructure*. The endocortical region exhibits CCCB in the posterior area, which decreases its thickness towards the lateral and posterior side. Presence of REVC is identified. In the lateral area, PFB and the presence of REVC are observed. The medial area presents PFB with PVC (Fig 6C). In the anterior area, CCCB and FLB are identified with the presence of SO and REVC (Fig 6D).

In the intracortical region, the posterior area shows CCCB, the medial area presents FLB, and REVC are observed around the entire region (Fig 6E). The lateral area presents CCCB and PFB with REVC. The medial area presents PFB with the presence of PVC. The anterior area presents FLB with the presence of SO, towards the extreme edge in this area RVC is identified.

The pericortical region presents LB forming EFS around the entire section, although composed of bands of different thicknesses. Towards the middle of the posterior area, PFB with SO and LB is observed (Fig 6E).

#### Metacarpal III

*Bone microanatomy*. The metacarpal III of the individual 1 was analysed (Table 1, Fig 6B). The cross-section is triangular with rounded borders.

*Bone microstructure*. The endocortical region is characterised by the presence of PFB around the medullary cavity and RVC (Fig 6F). Resorption cavities are present in the posterior side.

The intracortical region is defined by presenting FLB with randomly distributed SO. These are more abundant in the posterior side compared to the rest of the section. PO are observed in the posterior, lateral and medial sides. The intracortical region also presents VC. The anterior, medial and lateral sides present bands of LB (annuli) and PFB with SO (Fig 6G). The bands are thicker in the lateral and posteromedial sides compared to the rest of the section (Fig 6B, 6F and 6G).

The pericortical region presents bands of LB and PFB covering almost the entire external surface of the cross-section, except for a small area of the posterior side where FLB is observed (Fig 6F and 6G).

#### First phalanges (forelimb)

**Fig 7:**
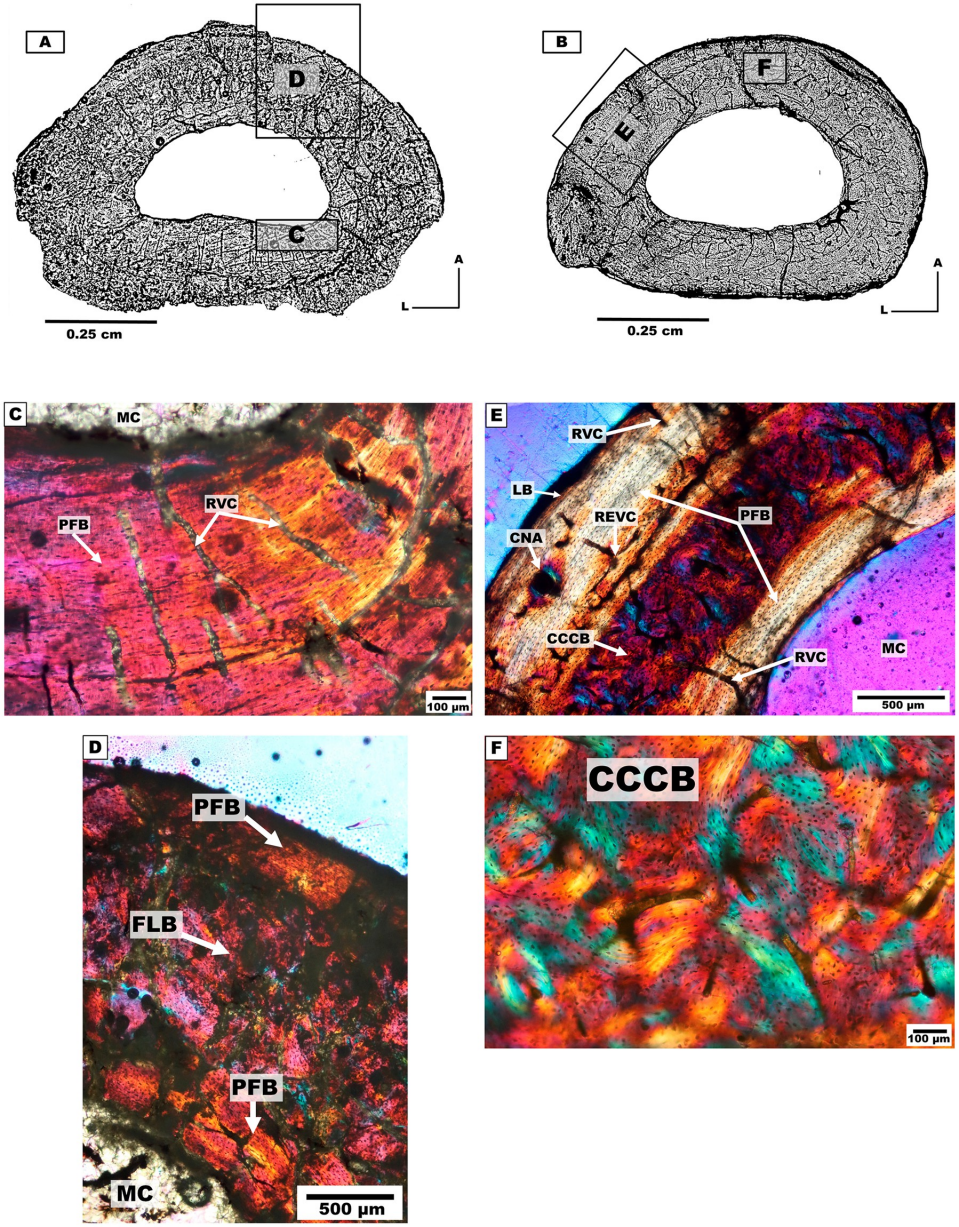
First phalanges cross-sections of the mesotheriine notoungulate *Caraguatypotherium munozi* from middle Miocene of Caragua, Huaylas formation. (A) First phalange cross-section for individual 1 (GEOUACH.HS.PFI.1); (B) First phalange cross section for individual 2 (GEOUACH.HS.PFD.1); (C) Endocortical region presence PFV and RVC can open to the medullary cavity (GEOUACH.HS.PFI.1); (D) FLB in the intracortical region and PFB in the pericortical region (GEOUACH.HS.PFI.1); (E) Diversity of structures in sections, PFB with RVC in the endocortical region, CCCB in the intracortical region, LB and PFB in the pericortical region with presence of RVC and CNA (GEOUACH.HS.PFD.1); (F) CCCB in the intracortical region (GEOUACH.HS.PFD.1). For abbreviations refer to Fig 4.

*Bone microanatomy*. Two first phalanges were studied, one from individual 1 and another from individual 2 (Table 1, Fig 7A and 7B). The cross-sections are sub-triangular, and present an open medullary cavity with the same morphology of the cross-section.

*Bone microstructure*. A conspicuous band of LB and PFB surrounds the entire medullary cavity, which is thicker in the posterior side. RVC are open to the medullary cavity and are more abundant in the posterior side (Fig 7C and 7E). The intracortical region presents FLB and CCCB, and isolated SO (Fig 7D–7F).

The pericortical region shows PFB and LB (Fig 7C and 7E), and RVCs appeared open to the external surface only in the phalange of individual 2 (Fig 7E).

#### Femur

**Fig 8:**
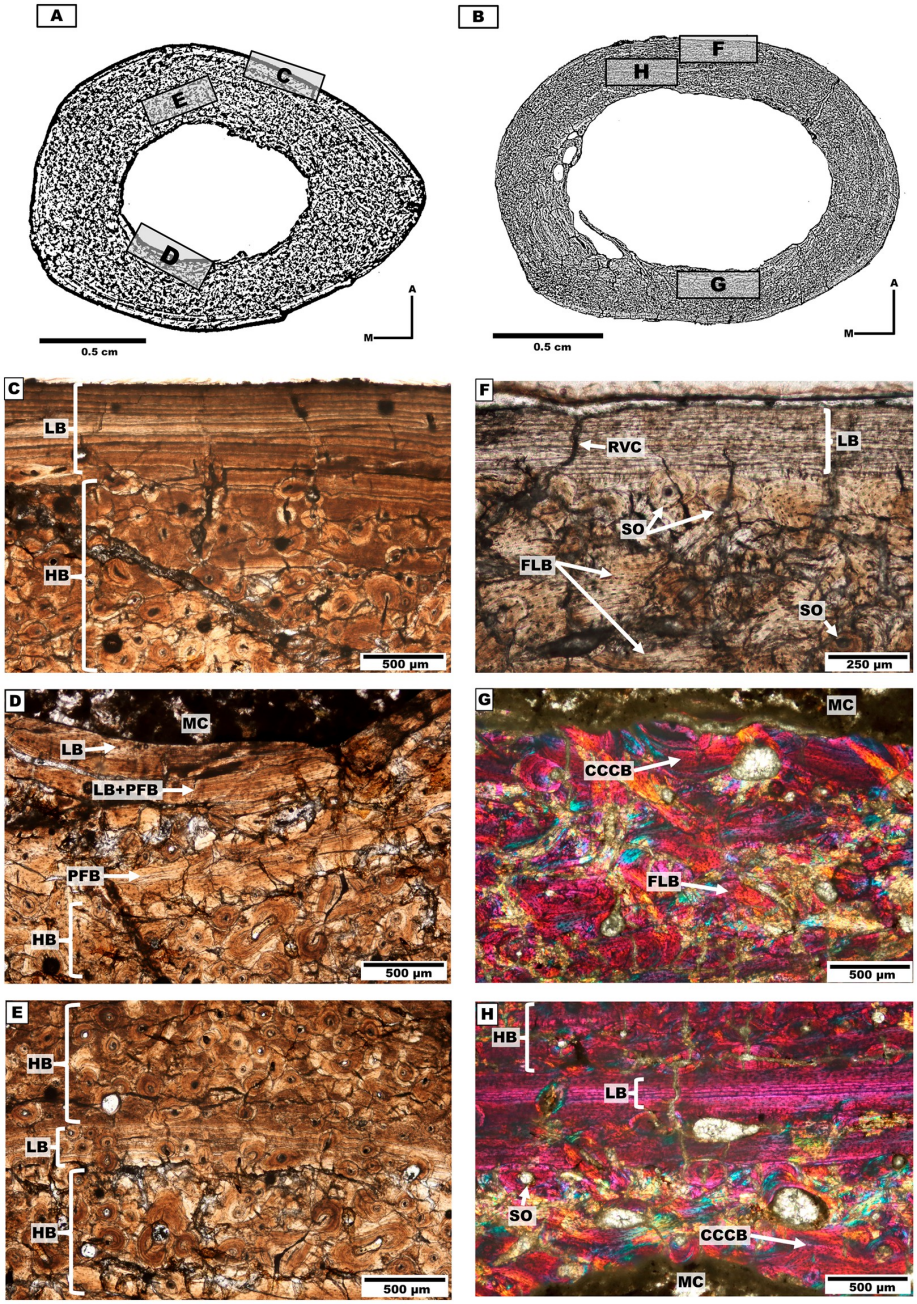
Femurs cross-sections of the mesotheriine notoungulate *Caraguatypotherium munozi* from middle Miocene of Caragua, Huaylas formation. (A) Femora cross-section for individual 5 (GEOUACH.HS.FI.2); (B) Femora cross-section for individual 7 (GEOUACH.HS.FD.1); (C) Pericortical region presence of LB and intracortical region presence of HB (GEOUACH.HS.FI.2); (D) LB and PFB in the Endocortical region (GEOUACH.HS.FI.2); (E) In the centre of intracortical region presence of LB between HB (GEOUACH.HS.FI.2); (F) Pericortical region, presence of LB in the external surface, FLB in the intracortical region and SO (GEOUACH.HS.FD.1); (G) CCCB in the endocortical region and FLB (GEOUACH.HS.FD.1); (H) In the centre of intracortical region presence of LB between HB (GEOUACH.HS.FD.1). For abbreviations refer to Fig 4.

*Bone microanatomy*. Two femora, of individual 5 and 7 were studied (Table 1, Fig 8A and 8B). The cross-section has a sub-rectangular morphology (mediolaterally elongated), with rounded borders and thick cortical walls. An open and ellipsoidal medullary cavity was present with no trabecular formation.

*Bone microstructure*. The endocortical region in the anterior, lateral and medial areas presents PFB and LB (Fig 8D), while the posterior area presents CCCB and scarce SOs (Fig 8G and 8H).

The intracortical region shows development of HB, although the interstitial primary matrix of FLB was still noticeable (Fig 8E–8H). The anterior region shows LB strongly stratified (Fig 8C and 8H). The intracortical region was almost completely remodelled with HB tissue, and only some RVC were discernible. The pericortical region is stratified showing intercalated bands of LB (Fig 8C and 8F).

#### Tibia

**Fig 9:**
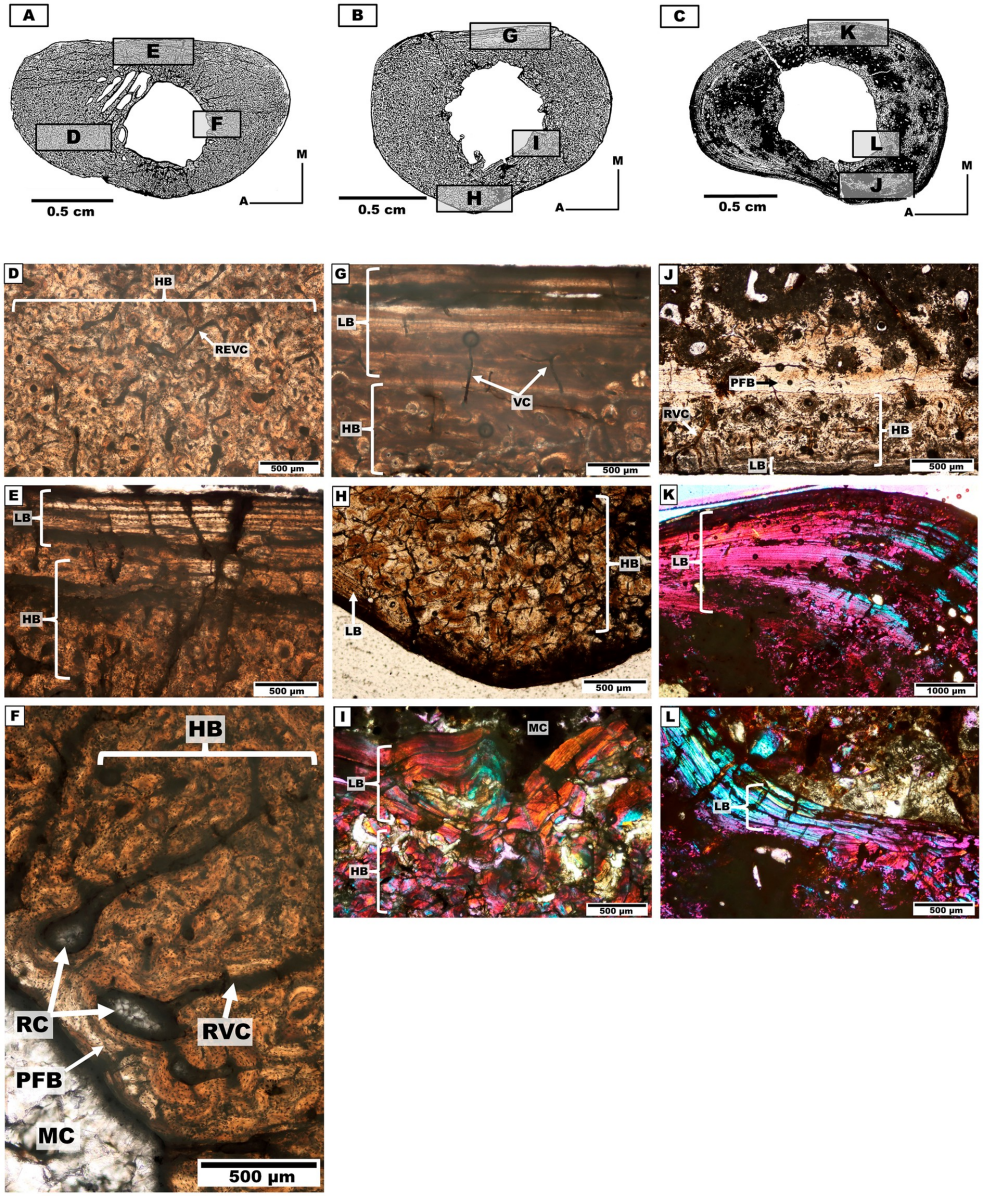
Tibia cross-sections of the mesotheriine notoungulate *Caraguatypotherium munozi* from the middle Miocene of Caragua, Huaylas formation. (A) Tibia cross-section for individual 4 (GEOUACH.HS.TI.1); (B) Tibia cross-section for individual 5 (GEOUACH.HS.TI.2); (C)Tibia cross section for individual 6 (GEOUACH.HS.TI.3); (D) HB in the intracortical region and presence of REVC (GEOUACH.HS.TI.1); (E) LB in the pericortical region and HB in the intracortical region (GEOUACH.HS.TI.1); (F) PFB in the endocortical region and RC (GEOUACH.HS.TI.1); (G) LB in the pericortical region, presence of VC and HB in the intracortical region (GEOUACH.HS.TI.2); (H) HB in the intracortical region; LB in the pericortical region (GEOUACH.HS.TI.2); (I) LB in the endocortical region and HB in the endocortical region (GEOUACH.HS.TI.2); (J) LB in the pericortical region, PFB in the interstitial spaces in the endocortical region (GEOUACH.HS.TI.3); (K) LB in the pericortial region and intracortical region (GEOUACH.HS.TI.3); (L) LB in the endocortical region (GEOUACH.HS.TI.3). For abbreviations refer to Fig 4.

*Bone microanatomy*. Three tibiae from individuals 4, 5 and 6 were described (Table 1, Fig 9A–9C). The cross-sections have an ellipsoidal shape with sharper pronunciation in the lateral region.

*Bone microstructure*. The endocortical region shows PFB and LB surrounding the medullary cavity. Open to the medullary cavity appear RVC and sometimes associated with resorption cavities present in the region (Fig 9F, 9G and 9L).

The intracortical region shows a high degree of bone remodelling with formation of HB (Fig 9D–9J), although the interstitial spaces of a primary matrix corresponding to PFB are still observed in some areas (Fig 9J). The pericortical region shows a band of LB (EFS) which is increased in width in the medial side (Fig 9E, 9G, 9K).

#### Metatarsal II

**Fig 10:**
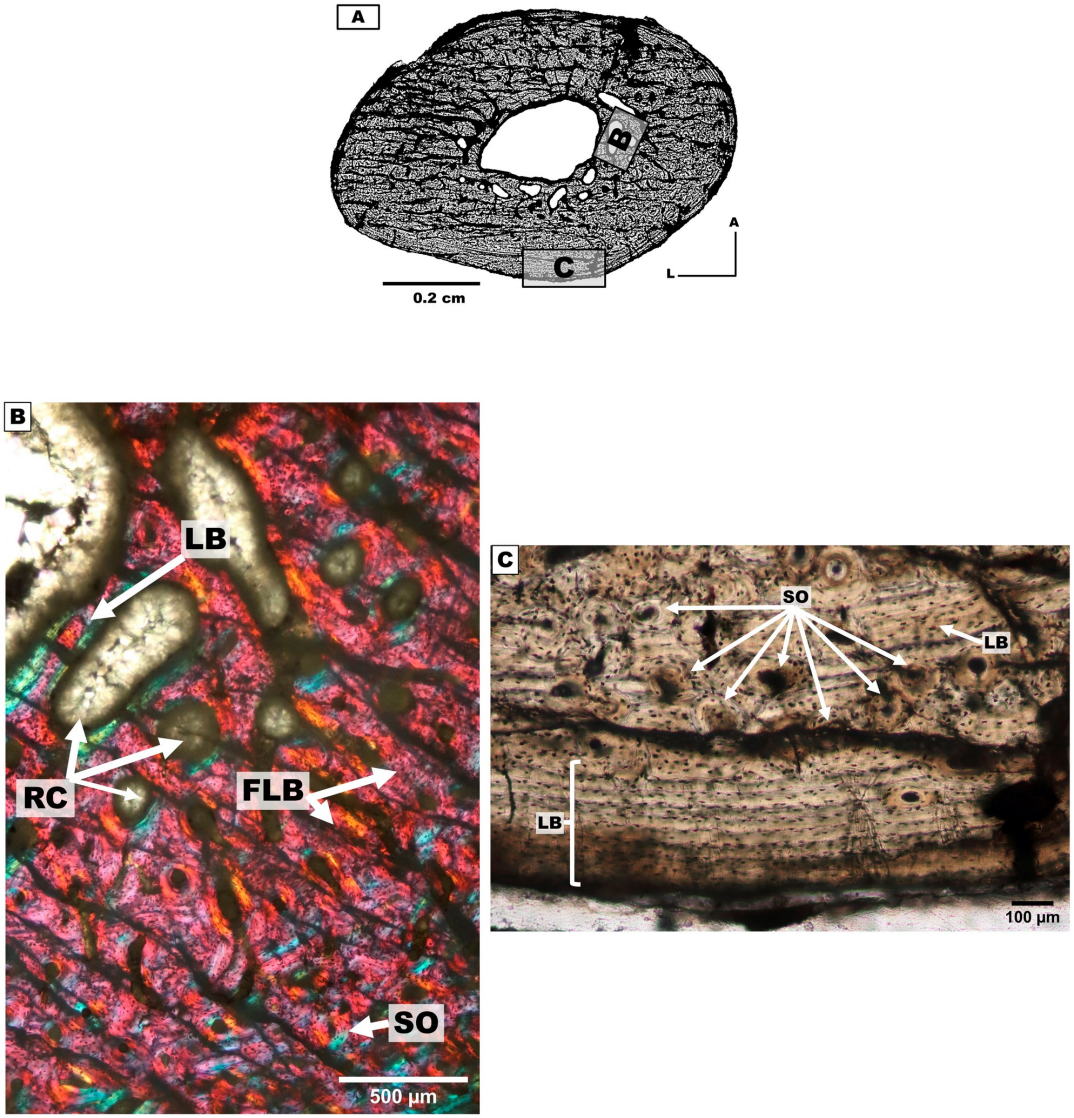
Metatarsal II cross-sections of the mesotheriine notoungulate *Caraguatypotherium munozi* from the middle Miocene of Caragua, Huaylas formation. (A) Metatarsal cross-section for individual 4 (GEOUACH.HS. MTSI.1); (B) RC and LB in the endocortical region, presence of SO in the endocortical region and FLB in the interstices (GEOUACH.HS. MTSI.1); (C) LB in the pericortical region, SO in the intracortical region (GEOUACH.HS. MTSI.1). For abbreviations refer to Fig 4.

*Bone microanatomy*. One metatarsal II from individual 4 was analysed (Tables 1–3, Fig 10A). The cross-section is ellipsoidal and contains a rounded medullary cavity with the presence of trabeculae.

**Table 2:** Histological structures identified in previous studies where notoungulate remains were used.

Bone microstructure	*Nesodon taweretus* (Toxodontidae),MHNSRP-PV 1010 and MHNSRP-PV 1008 [36]	*Protypotherium* sp. (Interatheriidae),YM-PU15341 and YM-PU15825 [18]		*Plesiotypotherium achirense* (Mesotheriidae), MNNHN.F.ACH 18; MNHN.F.ACH 34 and MNHN.F.ACH 46 [18]		*Meostherium* sp. (Mesotheriidae) (Pencil number: 273, ink label: 1938–583. Anat. Comp. 1878) [31]	*Toxodon* sp. (Toxodontidae) (Ink label: Anat. Comp. 1878) [31]	*Toxodon platensis* (Toxodontidae),CTES-PZ 1564 and CTES-PZ 1595 [34]	*Paedotherium bonaerense* (Hegetotheriidae), MD-FM-13-44 [35]	*Thomashuxleya externa* (Isotemnidae), MPEF-PV 8166 [37]
	Ulna	Humerus	Femur	Humerus	Femur	Ulna	Ulna	Ribs	Hemimandible	Femur
Compact bone	X	X	X	X	X	X		X	X	X
Compacted coarse cancellous bone	X							X	X	X
Woven bone										X
Parallel fibered bone	X									
Lamellar bone	X									X
Osteocyte lagoons	X							X	X	
Vascular canal	X						X		X	X
Primary osteon						X			X	
Haversian system	X					X	X	X		X

**Table 3:** Histological structures in the appendicular skeleton of *Caraguatypotherium munozi*. Abbreviations in Fig 4.

Appendicular skeleton/Histological structures	CCCB	FLB	PFB	LB	LTB	HB	VC	RVC	REVC	PVC	SO	PO
Humerus	X	X	X	X		X	X	X	X		X	X
Ulna	X	X	X	X			X	X	X	X	X	X
Metacarpal III		X	X	X			X				X	X
1^st^ Phalanges	X	X	X	X				X				
Femur	X		X	X	X	X		X			X	
Tibia		X	X	X		X	X	X				
Metatarsal II		X		X		X		X			X	

*Bone microstructure*. The endocortical region shows LB surrounding the whole medullary cavity. The posterior, medial and lateral sides present enlarged vascular canals (RVC) under resorption (Fig 10B).

The intracortical region presents FLB with development of SOs in the medial and posteromedial sides, and extending towards the pericortical region. The rest of the section (anterior and lateral sides) did not exhibit considerable remodelling or HB (Fig 10B and 10C).

The pericortical region exhibited LB (EFS) of variable thickness around the bone surface, which is wider in the posterior side and thinner in the lateral side (Fig 10C).

#### Individual 1 (HUAY15-100)

**Fig 11:**
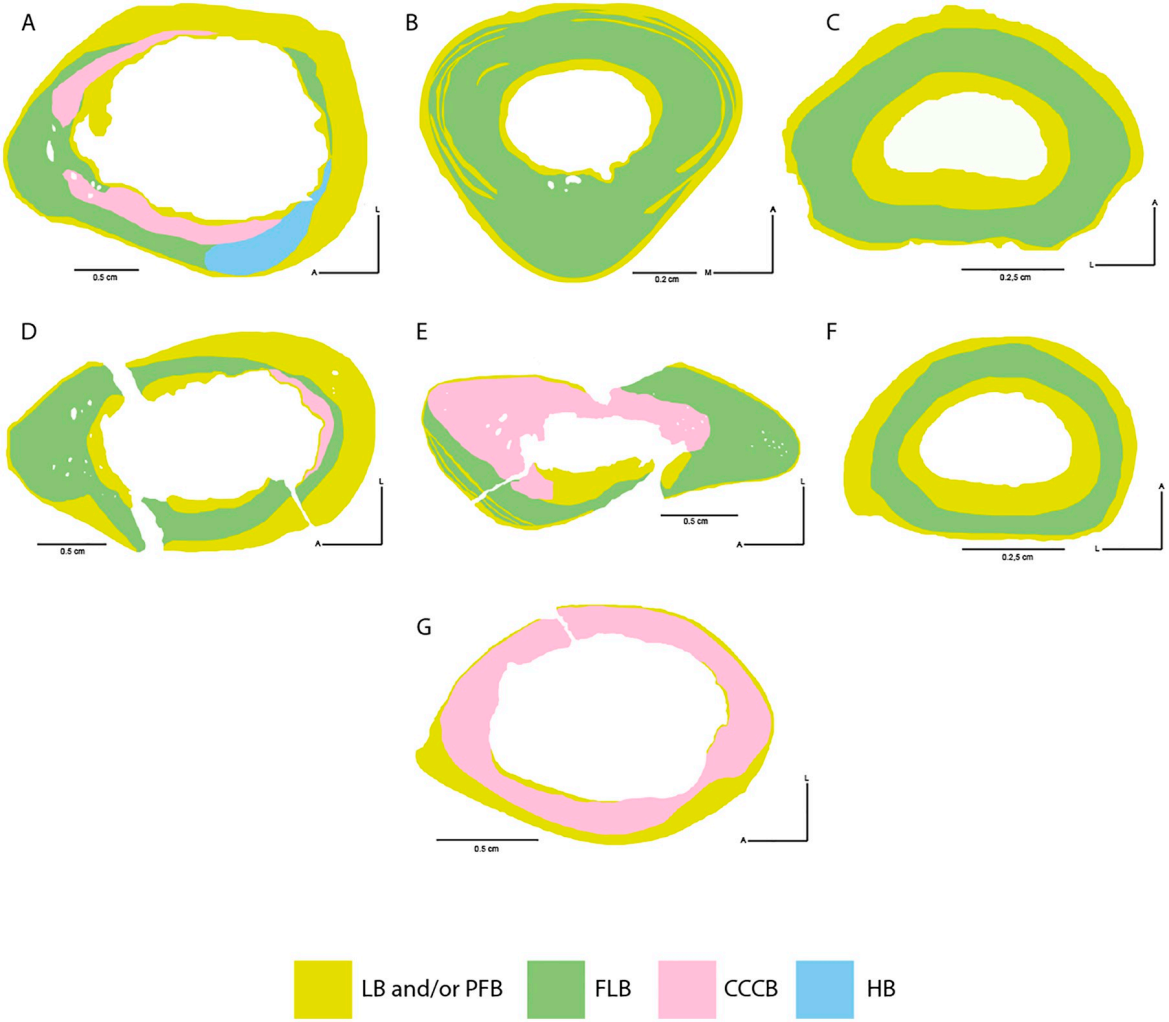
Types of bone tissue in section of *Caraguatypotherium munozi* from the middle Miocene of Caragua, Huaylas formation. Individual 1 (HUAY15-100): A, B and C, Individual 2 (HUAY 15–200): D, E and F, Individual 3 (HUAY17-05): G. (A) Left humerus (GEOUACH.HS.HI.1); (B) Left Metacarpal III (GEOUACH.HS.MTC.1); (C) 1^st^ left phalange (GEOUACH.HS.PFI.1); (D) Right humerus (GEOUACH.HS.HD.1); (E) Right ulna (GEOUACH.HS.UD.1); (F) 1^st^ left phalange (GEOUACH.HS.PFD.1); (G) Left humerus (GEOUACH.HS.HI.2). For abbreviations refer to Fig 4.

Considering the histological description made for the left humerus (GEOUACH.HS.HI.1; Fig 11A), it is possible to infer a rapid growth (presence of FLB) in the anterior and anterolateral side. Predominance of LB is observed, although the medial side shows clear intracortical remodelling. In the lateral side, FLB is observed in the intracortical region, with some degree of remodelling (SO). EFS is observed in the pericortical region.

In the posterior side, the EFS is extensive, almost covering the entire cortex, with isolated SO.

The sample corresponding to the left metacarpal III (GEOUACH.HS.MTC.1; Fig 11B) allowed identification of FLB tissue in the intracortical region, which is alternated with slow growing bone types in the anterior, lateral and medial sides, thus forming zonal bone. This information enables us to infer a temporal restriction of growth, as well as cortical drift towards the posterior region of the element. Additionally, the identification of EFS in the pericortical region of the whole section, clearly suggests a decreased rate of growth (Fig 11B).

The first left phalange (GEOUACH.HS.PFI.1; Fig 11C) provides evidence of a relatively uniform growth around the cortex, and the maintenance of a homogeneous structure throughout the bone element. LB with a high degree of vascularization was also present surrounding the medullary cavity. Additionally, in the intracortical region FLB deposition was identified indicating rapid growth of the cortex, followed by the development of an EFS, thus suggesting a slow rate of growth (Fig 11C).

The humerus presents less deposition of PFB and LB in the pericortical region as compared with the more distal elements, indicating that this bone may have reached skeletal maturity later than the more distal elements, and therefore growing for a longer period of time during life. Nevertheless, bone remodelling appears more extensive in the cortex of the humerus samples in comparison to the more distal elements (metacarpal and phalange), which are scarcely remodelled. Thus, we infer that bone remodelling is present in more proximal elements of the forelimb (humerus). Overall, the forelimb bones of the individual 1 exhibited relatively mature bone histology evidenced by deposition of EFS in pericortical regions, which suggests slow somatic growth rates at the time of death.

#### Individual 2 (HUAY15-200)

The histological structures of the right humerus (GEOUACH.HS.HD.1; Fig 11D) provided evidence supporting that the anterior side of the bone was under active growing at the time of death based on the presence of tissue with high rate of bone deposition and high degree of vascularisation.

The posterior side of the humeral section showed preservation of the humeral anatomy based on the presence of LB and PFB, a large area of the cortex allowing the retention of the elliptical shape of the element.

The lateral and medial sides presents similar histological structures characterised by having an intracortical region of FLB, exhibiting towards the posterior side LB. Based on this information, it can be inferred that the ontogenetic growth of the humerus would correspond with faster and constant growth in the anterior side (near the dectopectoral crest) contrasting with the slow growing tissues present towards the lateral, medial and posterior sides (Fig 11D).

The growth pattern of the ulna (GEOUACH.HS.UD.1; Fig 11E) is characterised by a deposit of fast-growing tissues in the anterior and posterior sides of the section, contrasting with the presence of low-growing tissue in the lateral and medial sides. This is in agreement with the shape of the analysed section, where higher length to width rate predominates (Fig 11E).

The histological features of the sample, corresponding to the first right phalange (GEOUACH.HS.PFD.1; Fig 11F), allow the identification of FLB and EFS in the pericortical region of the sample. The specimens also shows a high degree of vascularisation with VC opened to the cortical layer. The posterolateral side of the section shows cortical drift characterised by resorption from the medullary cavity and the deposition of periosteal LB to the lateral side of the section (Fig 11F).

The forelimb bones of the individual 2, shows predominance of FLB in intracortical regions, with some deposition of EFS in pericortical region. Additionally, all the samples presents high degree of vascularisation with VC opened to the cortical layer. According ly, the presence of EFS indicates that the individual has reached their somatic maturity (Fig 11D–11F).

#### Individual 3 (HUAY17-05)

The left humerus sample (GEOUACH.HS.HI.2; Fig 11G) from individual 3 corresponds to the smallest in size of the other two humeri analysed in this study (GEOUACH.HS.HI.1; and GEOUACH.HS.HD.1). The cortex is characterised by the presence of CCCB. In the anterolateral, lateral and posterior sides of the section, FLB is observed in smaller quantities than the CCCB. The presence of EFS in the humerus section shows that the individual had reached a state of somatic maturity, although other areas were still in the process of bone modelling (Fig 11G).

#### Individual 4 (HUAY16-100)

**Fig 12:**
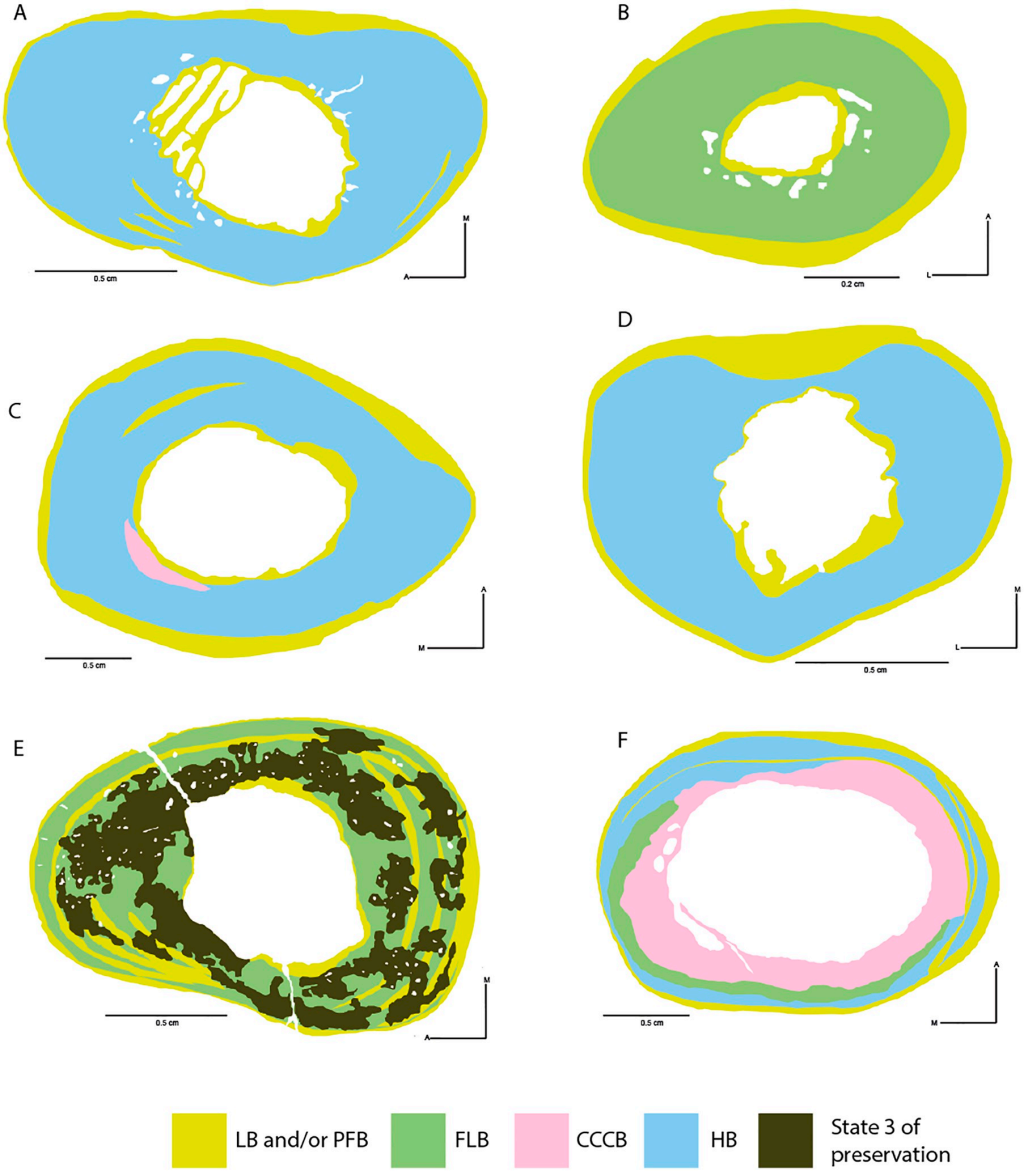
Types of bone tissue in section of *Caraguatypotherium munozi* from the middle Miocene of Caragua, Huaylas formation. Individual 4 (HUAY16-100): A and B, Individual 5 (HUAY15-84): C and D, Individual 6 (HUAY15-027): E, Individual 7 (HUAY17-02): F. (A) Left tibia (GEOUACH.HS.TI.1); (B) Left metatarsal II (GEOUACH.HS. MTSI.1); (C) Left femur (GEOUACH.HS.FI.2); (D) Left tibia (GEOUACH.HS.TI.1); (E) Left tibia (GEOUACH.HS.TI.3); (F) Right femur (GEOUACH.HS.FD.1). For abbreviations refer to Fig 4.

The analysed bone elements from individual 4 correspond to appendicular elements of the left hindlimb. The sample of the left tibia (GEOUACH.HS.TI.1; Fig 12A) shows signs of remodelling in nearly the entire section. We identified a primary matrix corresponding to alternating bands of LB and PFB bone in the lateral side and formation of EFS. The trabecular bone in the medullary cavity indicates predomination of bone resorption (and cortical drift) towards the anterior side (Fig 12A).

In the metatarsal II (GEOUACH.HS. MTSI.1, Fig 12B), EFS is identified in the entire pericortical region maintaining similar thickness throughout the section. In the intracortical region, it is possible to observe FLB with resorption cavities in the endocortical region.

Overall, the hindlimb bones of the individual 4 show EFS, and no apparent active osteogenesis. The tibia exhibited showed considerably more remodelling than the metatarsal II (Fig 12A and 12B).

#### Individual 5 (HUAY15-084)

The tibia (GEOUACH.HS.TI.2, Fig 12C) and femur (GEOUACH.HS.FI.2, Fig 12D) of individual 5 showed a high degree of bone remodelling, although pericortical margins showed clear development of EFS. The anterior aspect of the femur showed zonal bone with intercalations of LB, suggesting a slowly and cyclical bone growth at the time of death. The tibia shows comparatively more remodelling than the femur, and the EFS appears wider in the medial side and narrower in the lateral side compared to the rest of the section (Fig 12D).

It can be concluded that these bone elements have reached an advanced stage of development where the rate of bone deposition is low, with inactive periosteal surfaces and a high degree of bone remodelling throughout the cortex.

#### Individual 6 (HUAY15-027)

Considering the intermediate and poor degree of preservation (state 2 and 3) of the tibia (GEOUACH.HS.TI.3, Fig 12E), it was possible to identify a pericortical region presenting EFS, which is represented by a thicker and more notorious band in the medial region. Relatively fast growing tissues were observed in the lateral, anterior and posterior regions, as well as presence of vascular canals opening towards the external surface of the bone (Fig 12E). Some patches of primary bone tissue (FLB) were identified in the intracortical region. These observations allow us to infer that this individual was still in an active stage of bone formation at the time of death.

#### Individual 7 (HUAY17-02)

The femur (GEOUACH.HS.FD.1, Fig 12F) presents a histology characterised by proportional growth (similar thicknesses) in all regions of the bone cortex, and the presence of FLB in the intracortical region. An EFS is observed around the entire pericortical region.

Between the intracortical and pericortical regions, zonal bone with intercalations of LB were observed, which suggested that this bone was deposited slowly and cyclically (Fig 12F). Accordingly, we conclude that at the time of death, the femur was in an advanced stage of somatic maturity.

### Humeral growth

Through the comparison of the three analysed humeri from specimens 1 to 3, it was possible to determine the ontogenetic growth of C. munozi. Thus, the humeral growth in this animal has been divided in two ontogenetic stages (Fig 11A, 11D and 11G).

1. A subadult stage corresponds to a specimen presenting a humerus with fused epiphyses. This stage is characterised by the absence of lamellar bone in the entire endocortical region. In the intracortical region there is a predominance of CCCB, characteristic of bone elements with a low degree of resorption, as well as FLB with VREC. The pericortical region presents a predominance of EFS, which is observed as a thin band only in the anteromedial, medial and posteromedial areas. In addition, there are radial vascular channels that open to the outside. This is shown in GEOUACH.HS. HI.2 (Fig 11G).
2. An adult ontogenetic stage corresponds to a specimen whose humerus presents fused epiphysis. With LB in the endocortical region covering the borders of the medullary cavity. The intracortical region is characterised by the predominance of the cortex with FLB and CCCB. In the pericortical region,EFS is present and it is thicker in the posterior area. These structures may be accompanied by reticular vascular canals opening to the exterior in some cases. This ontogenetic stage is shown in GEOUACH.HS.HI1 from individual 1 and GEOUACH.HS.HD1 from individual 2 (Fig 11A and 11D respectively).

## Discussion

Ontogenetic reconstructions of mammalian species based on paleohistological assessments are scarce, principally due to the usually limited number of individuals available for this kind of analysis. This is particularly pronounced for the order Notoungulata, from which only brief descriptions of their bone microstructure have been reported [18, 28, 31–37] (Table 2).

**Fig 13:**
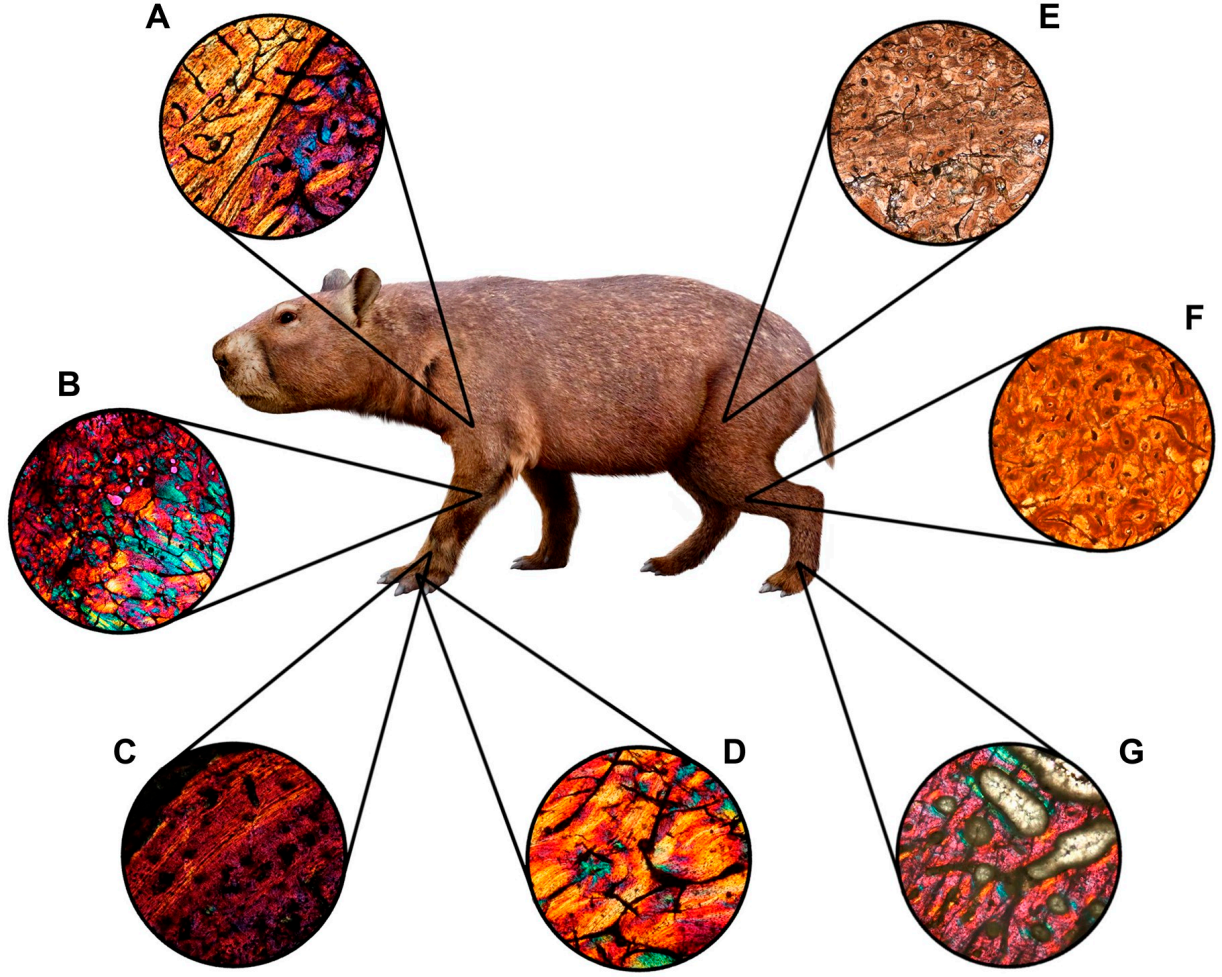
Reconstruction in life of *Caraguatypotherium munozi* from the middle Miocene of Caragua, Huaylas formation and histological detail. (A) Humerus; (B) Ulna; (C) Metacarpal III; (D) 1^st^ Phalange; (E) Femur; (F)Tibia; (G) Metatarsal II. (Illustration made by Carlos Andrés Espinosa Bustos).

In this study, we evidenced the highly diverse bone microstructure of *C*. *munozi* (Table 3, Fig 13). The distribution and amount of bone tissues within the cortex showed high variation between different appendicular elements and even within the same bone, as well as during ontogeny. This indicates that the development of the long bones of *C*. *munozi* involves a complex process of bone modelling and remodelling, but also that bone elements within the same individual and sections growth at different rates. In general, our results confirm the presence of some bone tissues reported previously for mesotheriids [18, 31, 35], and present several previously undocumented tissue types to this group, thus suggesting that the histodiversity of mesotheriids is as rich as that of other extant mammals (e.g. [64, 65, 68, 74, 76, 77]).

Kolb et al. [28] made a preliminary “characterisation” of the paleohistology of Notoungulata, which have been described as presenting bones with a compacted, well vascularised cortex and mostly longitudinal vascular canals. Additionally, histological features in Notoungulata include the presence of few canals of regular orientation, large and abundant osteocyte lacunae, along with the identification of HB in the toxodontids *Toxodon and Nesodon*, as well as the mesotheriid *Mesotherium*. Surrounding the primary matrix, a tissue composed of LB and PFB bone is described, along with localised areas of woven bone characterised by rounded osteocyte lacunae; and an outer cortex layer composed of PFB with few SO in clear contrast to the highly remodelled inner cortex [26].

For mesotheriids, the presence of HB [31], and PO and SO have only been previously described for *Mesotherium* sp. [18, 31]. Therefore, our investigation provides novel information for this group based on the histological variability of *C*. *munozi* (Table 3).

Thus, we reported for the first time in Mesotheriidae the presence of five different bone types: FLB, PFB, LB, HB, and CCCB [70, 71, 78, 79]. In addition to the description of vascularisation in previous studies (e.g. [28, 31, 34, 36, 37]), we include the presence of radial and reticular vascularisation, together with the plexiform orientation of the vascular canals.

The comparison between specimens used in this study and those of other notoungulates (lacking ontogenetic series) allowed us to have a better picture of the histological variability of the appendicular skeleton.

Interestingly, the presence of CCCB tissue, initially described by Enlow [70], has rarely been described in histological studies of fossil and extant mammals. The presence of this bone tissue has been described for young individuals [79], but in the case of the fossil remains from *C*. *munozi*, it is possible to find this type of tissue associated with EFS (Figs 11 and 12). Accordingly, it is inferred that CCCB remains in advanced ontogenetic stages of the animal. This process has also been observed in adult subterranean rodents (*Bathyergus suillus*: [68]) and adult aakvarks (*Orycteropus afer* [80]). Therefore, this tissue type is not exclusive to early ontogenetic stages, and the presence of this tissue in adulthood in *C*. *munozi* represents a remnant of the earlier endochondral bone formation and secondary compaction which was not obliterated during ontogeny, probably because a low bone resorption to maintain high levels of cortical thickening [68]. This would also suggest that the robust limb bones of *C*. *munozi* and other mesotheres that exhibit thick cortical walls in their long bones, might be adapted to increase the bending and torsional resistance experienced during activities of high impact, such as parasagittal scratch-digging (e.g. [68]). In this regard, some studies have suggested a certain degree of fossoriality in mesotheres [9].

Based on the results obtained, it is possible to support a significant size and histological variation of the forelimbs among sub-adult and adult individuals of *C*. *munozi*. This is important, especially considering that there is a strong size variation of individuals within close age ranges, that could lead to material identification to earlier ontogenetic stages based on macro-anatomic observations [42, 81, 82].

Additionally, our findings support that bone corresponding to younger stages of ontogenetic development in *C*. *munozi* present a structure that has already been described in other vertebrates [28, 67, 68, 80, 83]. This structure features a higher proportion of fast-growing microstructural bone elements in comparison with more advanced ontogenetic stages where slow-deposition of histological elements predominates.

Different studies have provided insights regarding the appendicular skeleton in mammals, pointing out that the best bone elements to be analysed are the tibia and femur, mainly because these elements exhibit low resorption and bone remodelling, so that the bone tissues developed early in ontogeny can still be observed [22, 30]. In contrast, we found that femur and tibia samples of *C*. *munozi* present high levels of bone remodelling in the entire cortex, indicating that these elements are not very informative for reconstructing the ontogeny of the animal.

Although in the present study is not possible to precise whether the hindlimbs of *C*. *munozi* reached skeletal maturity before the forelimbs (because the lack of hindlimbs and forelimbs of the same individual), our sample shows clearly a considerable difference growth patterns and microstructural arrangement between limbs, thus indicating a noticeable modular growth of the extremities. Such hypothesis should be tested using a larger and more complete dataset including individuals with both forelimbs and hindlimbs.

The *C*. *munozi* femur presents a stratified LB in the intracortical region. This marked stratification indicates a cessation and resumption of bone deposition in the bone element and may indicate a pause in the growth or even seasonality [21]. Similar growth interruption conformed by LB deposited in the intracortical region have also been described in fossil perissodactyls such as the equid hipparionini *Hipparion concudense*, probably indicating a cessation and a new resumption of growth in the animal [27]. It is not fully understood how exogenous and endogenous factors can influence the formation of this type of bone deposition. However, early and recent studies of extant taxa documenting cyclical growth in mammals have provided conclusive evidence that such variation in growth patterns are more widespread among mammals as previously thought. Köhler et al. [23] suggested that cyclical bone growth in mammals is a universal trait of homoeothermic endotherms and that the arrested growth observed during unfavourable seasons in endotherms may arise as part of a plesiomorphic thermo-metabolic strategy for energy conservation. The finding of growth marks and therefore cyclical growth in bone tissues of subterranean animals, such as African mole-rats (Bathyergidae) [68], the greater mole rat (Spalacidae) [84, 85] and moles (Talpidae) [86], which experience considerably lower levels of seasonality and environmental stress as compared to aboveground mammals supports this hypothesis, and suggests that environmental factors alone are not the only factor behind the process of cyclical growth. The finding of growth marks and cyclical bone growth in organisms from captive conditions such as naked mole-rats [64] and small primates [67], which experience lower levels of stress due to unlimited resources also support this hypothesis.

As previously described for other extinct animals [22, 28, 78, 79], the presence of ZB in *C*. *munozi* may represent the acquisition of a similar growth pattern in these extinct South American mammals, most likely evolved before the split from the rest of the mammalian lineages. Our analysis provides conclusive evidence for a highly complex pattern of bone modelling and remodelling in this taxon. It is important to mention that despite these growth patterns represent an endogenous aspect of the biology of mammals, other external factors such as environmental fluctuations and/or availability of food resources may also reinforce and/or be synchronized with growth. In this sense, increments of the rainfall patterns between 11–10 Ma have been recently interpreted from the stratigraphic record of Caragua and nearby localities [48, 53, 87–90]. This information suggests the onset of wetter conditions and ecosystem re-organization at the local and regional scales. A brief tectonic deformational pulse in the Precordillera and concomitant uplift of the Western Andean orogenic front has also been recorded during this lapse [48, 51, 53, 88, 89]. Causality between both factors has been invoked through observed changes along the stratigraphic sections, including base level re-accommodation, modifications in the sedimentary regimes and watershed architecture, increment in basement-derived components, and associated landscape incision, beginning around 12 Ma [48, 53, 91]. Rapid climatic changes can lead to marked seasonality [90] imposing long lasting stressors over species and/or faunal interactions, with possible ecological and physiologic effects on individuals that can be observed in their skeletal structures, such as malnutrition or fibrous osteodystrophy [92, 93]. Similar factors also may impact populations, including the disruption of trophic dynamics, negative density-dependent effects, or selective local extirpation/extinctions [94]. Further research is required addressing the ontogeny and physiology of *C*. *munozi* and the remaining faunal components of Caragua, to assess how the environmental changes could be reflected in its histological record.

On the other hand, taxonomic limitations could sustain an alternative explanation for the observed differences. During the early to late Miocene diverse localities in Chile and nearby Bolivia such as Chucal, Cerdas, Nazareno, Quebrada Honda and Achiri, documents the sympatric coexistence is documented of more than one (two to three) mesotheriine species [39, 95–99]. Body size differences between some of these species have been recognized, supporting generic-level taxonomic distinction (i.e., [97]). An underestimated interspecific diversity in Caragua’s mesothere assemblage cannot be ruled out completely, especially concerning some of the isolated postcranial remains. Although different studies suggest that interspecific variation in some mesotherids is greater than previously thought [38, 33], intraspecific differences tend to be considered as intergeneric or interspecific differences as has been previously discussed (i.e. [42, 82, 98]). Despite this, morphological conservatism between the Caragua’s samples and spatial proximity of the recovered individuals supports intraspecific ontogenetic disparity as the most plausible cause for such body size differences. New collections and detailed analysis of the morphological variability (and its histological correlation) within *Caraguatypotherium munozi* and other Miocene mesotheres are needed in order to understand the evolutionary ecology of mid-latitude faunas and its relationship with the proposed rapid environmental and climatic changes occurred in the Central Andes, as well as in the whole continent, during the Neogene. Future studies in this direction will reveal higher complexity in the Notoungulate fauna, which recent research (e.g. [11, 100]) such as the present one, are just starting to unveil.

## Conclusions

Here we present the most comprehensive paleohistological study of Notoungulata, providing a detailed descriptionof fore- and hindlimb of several individuals of the Mesotheriid *Caraguatypotherium munozi*, allowing a better understanding on the variety of histological structures shown by these animals. Results indicates high variability and growth of differences. In particular, humeri shows less bone resorption and remodelling favouring the study of ontogenetic stages, with all different bone types acquired throughout the life cycle of the animal. On the contrary, hindlimbs show excessive remodelling, erasing all early records. Based on these observations, the ontogenetic stage of individuals 4 to 7 (HUAY 16–100, HUAY 15–084, HUAY 15–027, HUAY 17–02) could not be determined, but they did reach somatic maturity on their hind limbs, which could be acquired in an early stage. Individuals 1 and 2 (HUAY15-100 and HUAY15-200) can be recognised as adults. Histological structures of bone deposition (high and low rates) are in agreement with proposed age ranges based on anatomical features (e.g. epiphyseal fusion). Although the humerus (GEOUACH.HI.2) of individual 3 (HUAY15-100) was expected to represent a juvenile or immature animal due to its small size (up to 40% smaller than the largest one) and external fibrous bones, instead it showed a slow rate of bone deposition indicating a cessation of growth, typical for a sub-adult or even an adult. The quantified low ontogenetic variability but higher size differences, suggests that exogenous factors could act as potential stressors associated with an unstable environment. This is supported by tectono-stratigraphic and isotopic evidence that shows rapid environmental and climatic changes during the beginning of the late Miocene associated with orogenic uplift and concomitant re-organization of the drainage systems along the western tectonic front of the Central Andes.

## Supporting information

S1 Fig Bone elements of individual 1 (HUAY15-100). A: Left humerus anterior view (GEOUACH.HS.HI.1); B: Left metacarpal anterior view (GEOUACH.HS.MTC.2); C: 3^rd^ left phalange anterior view (GEOUACH.HS.TFI.1). Histological section area is indicated with a rectangle in black.
(TIFF)

S2 Fig Bone elements of individual 2 (HUAY15-200). A: Rigth humerus posterior view (GEOUACH.HS.HD.1); B: Right ulna medial view (GEOUACH.HS.UD.1); C: Left femur anterior view (GEOUACH.HS.FI.1); D: 3^rd^ right phalange anterior view (GEOUACH.HS.TFD.1). Histological section area is indicated with a rectangle in black.
(TIFF)

S3 Fig Bone element of individual 3 (HUAY17-05). A: Left humerus anterior view (GEOUACH.HS.HI.2). Histological section area is indicated with a rectangle in black.
(TIFF)

S4 Fig Bone elements of individual 4 (HUAY16-100). A: Left tibia anterior view (GEOUACH.HS.TI.1); B: Left metatarsal anterior view (GEOUACH.HS.MTSI.1). Histological section area is indicated with a rectangle in black.
(TIFF)

S5 Fig Bone elements of individual 5 (HUAY15-084). A: Left femur anterior view (GEOUACH.HS.FI.2); B: Left Tibia anterior view (GEOUACH.HS.TI.2). Histological section area is indicated with a rectangle in black.
(TIFF)

S6 Fig A: Bone element of individual 6 (HUAY15-027). Left tibia anterior view (GEOUACH.HS.TI.3); B: Bone element of individual 7 (HUAY17-02), Right femur anterior view (GEOUACH.HS.FD.1). Histological section area is indicated with a rectangle in black.
(TIFF)

## Abbreviations

CTES-PZ: Corrientes-Paleozoología
HS: Histology
MD-FM: Museo Municipal de Ciencias Naturales “Carlos Darwin”, Punta Alta (Buenos Aires Province)
MHNSR-PV: Museo de Historia Natural de San Rafael, Vertebrate Paleontology Collection, San Rafael, Mendoza, Argentina
MNHN: Museum national d’Histoire naturelle, Paris, France
MPEF-PV: Museo Paleontológico Egidio Feruglio- Paleontolgía de Vertebrados, Trelew, Chubut, Argentina
UACH: Universidad Austral de Chile
XX: Type of bone element (e.g., HI: Left humerus; HD: Right humerus; UD: Right ulna; MTCI: Left metacarpal; PFI: 1^st^ left phalange; FI: Left femur; FD: Right femur; TI: Left tibia; MTSI: Left metatarsal; PFD: 1^st^ right phalange)
XX: Year of field work
Y: Number of sample elements
YPM: Yale Peabody Museum of Natural History, New Haven, Connecticut, USA Identification of individuals: HUAYXX-YY: HUAY: Huaylas
YY: Item number collected. Identification of histological specimens: GEOUACH.HS.XX. Y: GEO: Geology department
