# Supplementary figures and images for: Paleohistology of Caraguatypotherium munozi (Mammalia, Notoungulata, Mesotheriidae) from the early late Miocene of northern Chile: A preliminary ontogenetic approach
Source: PLoS One. 2023 Mar 16;18(3):e0273127. doi: 10.1371/journal.pone.0273127 (PMC10019713; doi:10.1371/journal.pone.0273127)

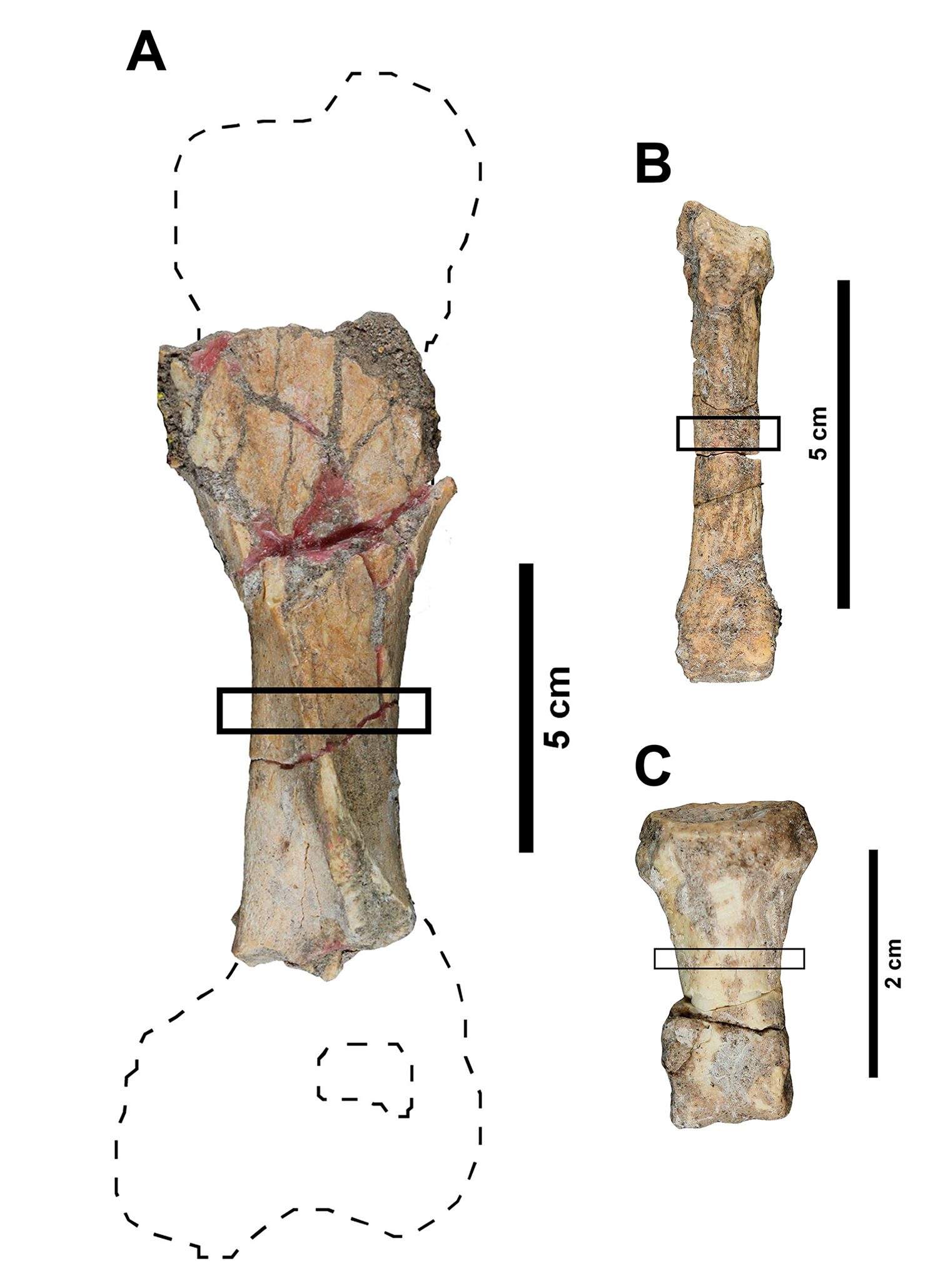

Supplement: S1 Fig — A: Left humerus anterior view (GEOUACH.HS.HI.1); B: Left metacarpal anterior view (GEOUACH.HS.MTC.2); C: 3rd left phalange anterior view (GEOUACH.HS.TFI.1). Histological section area is indicated with a rectangle in black. (TIFF) [file pone.0273127.s001.tiff]

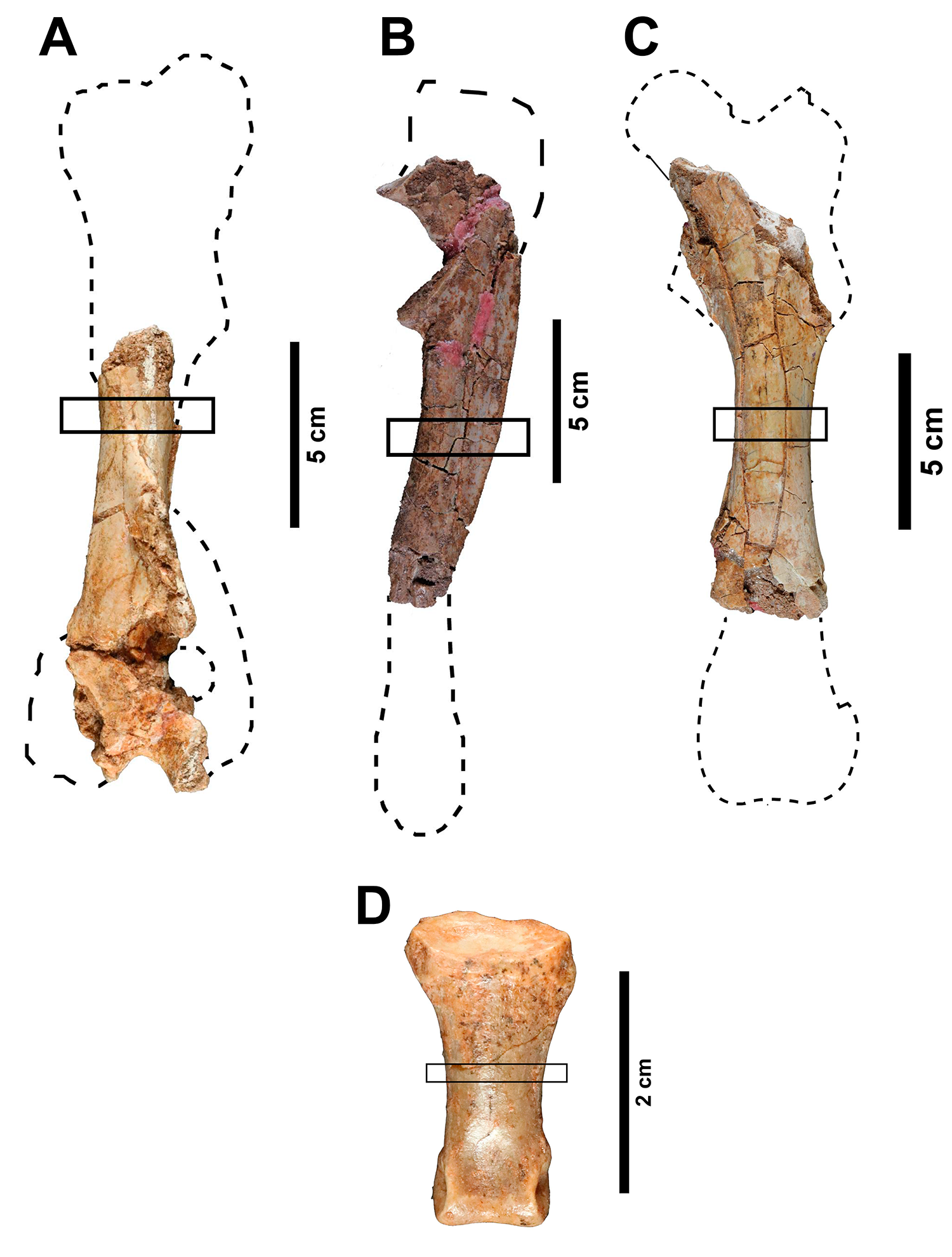

Supplement: S2 Fig — A: Rigth humerus posterior view (GEOUACH.HS.HD.1); B: Right ulna medial view (GEOUACH.HS.UD.1); C: Left femur anterior view (GEOUACH.HS.FI.1); D: 3rd right phalange anterior view (GEOUACH.HS.TFD.1). Histological section area is indicated with a rectangle in black. (TIFF) [file pone.0273127.s002.tiff]

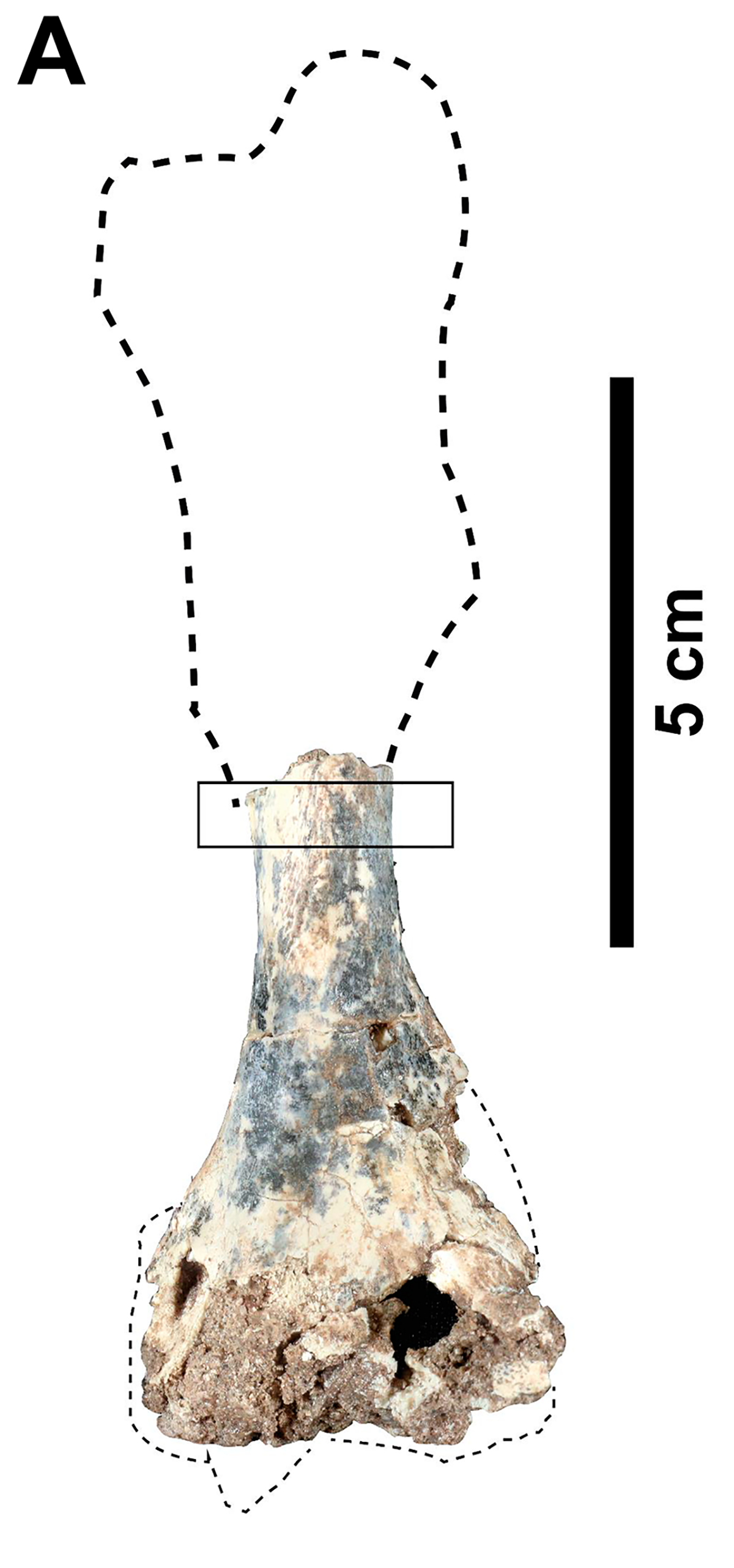

Supplement: S3 Fig — A: Left humerus anterior view (GEOUACH.HS.HI.2). Histological section area is indicated with a rectangle in black. (TIFF) [file pone.0273127.s003.tiff]

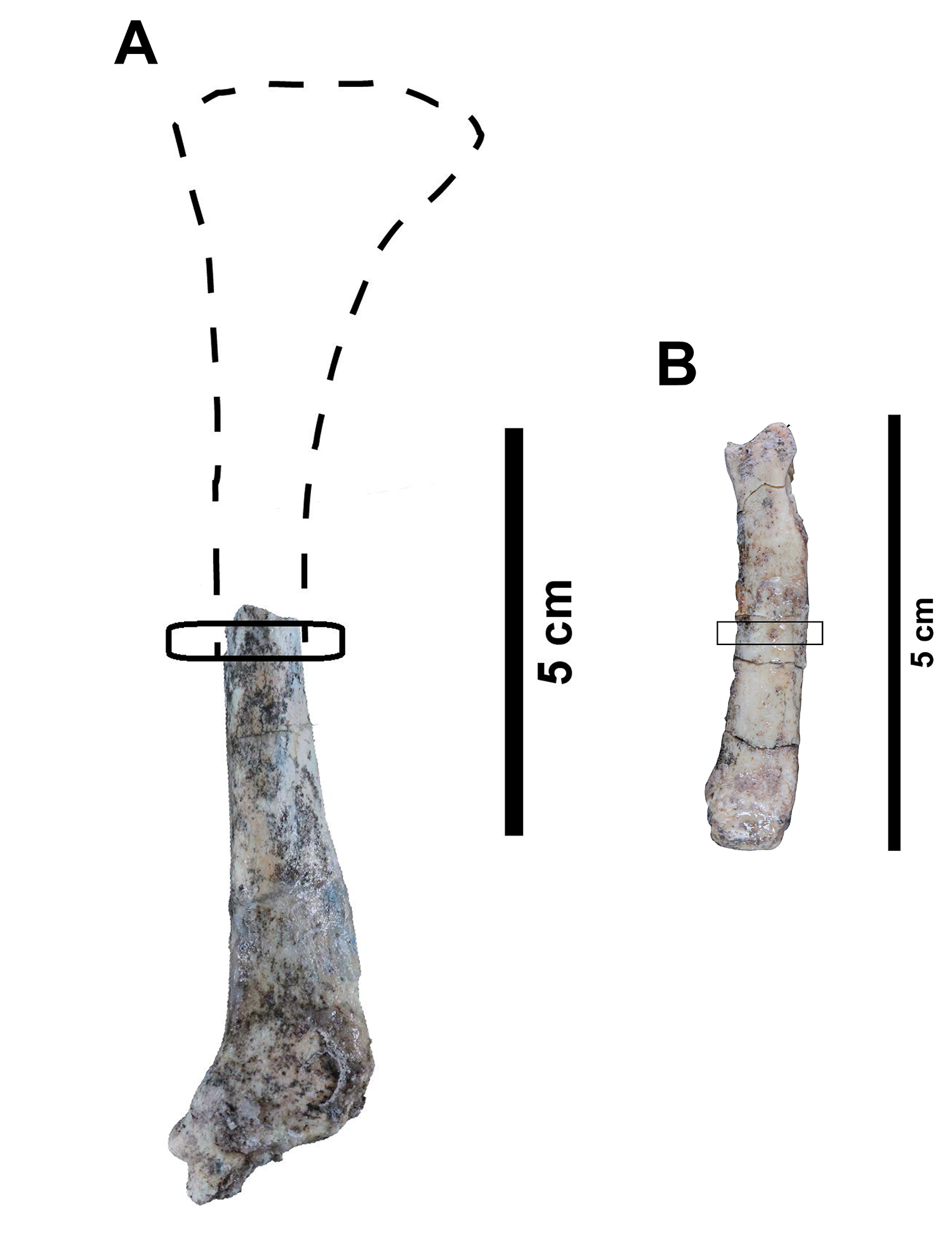

Supplement: S4 Fig — A: Left tibia anterior view (GEOUACH.HS.TI.1); B: Left metatarsal anterior view (GEOUACH.HS.MTSI.1). Histological section area is indicated with a rectangle in black. (TIFF) [file pone.0273127.s004.tiff]

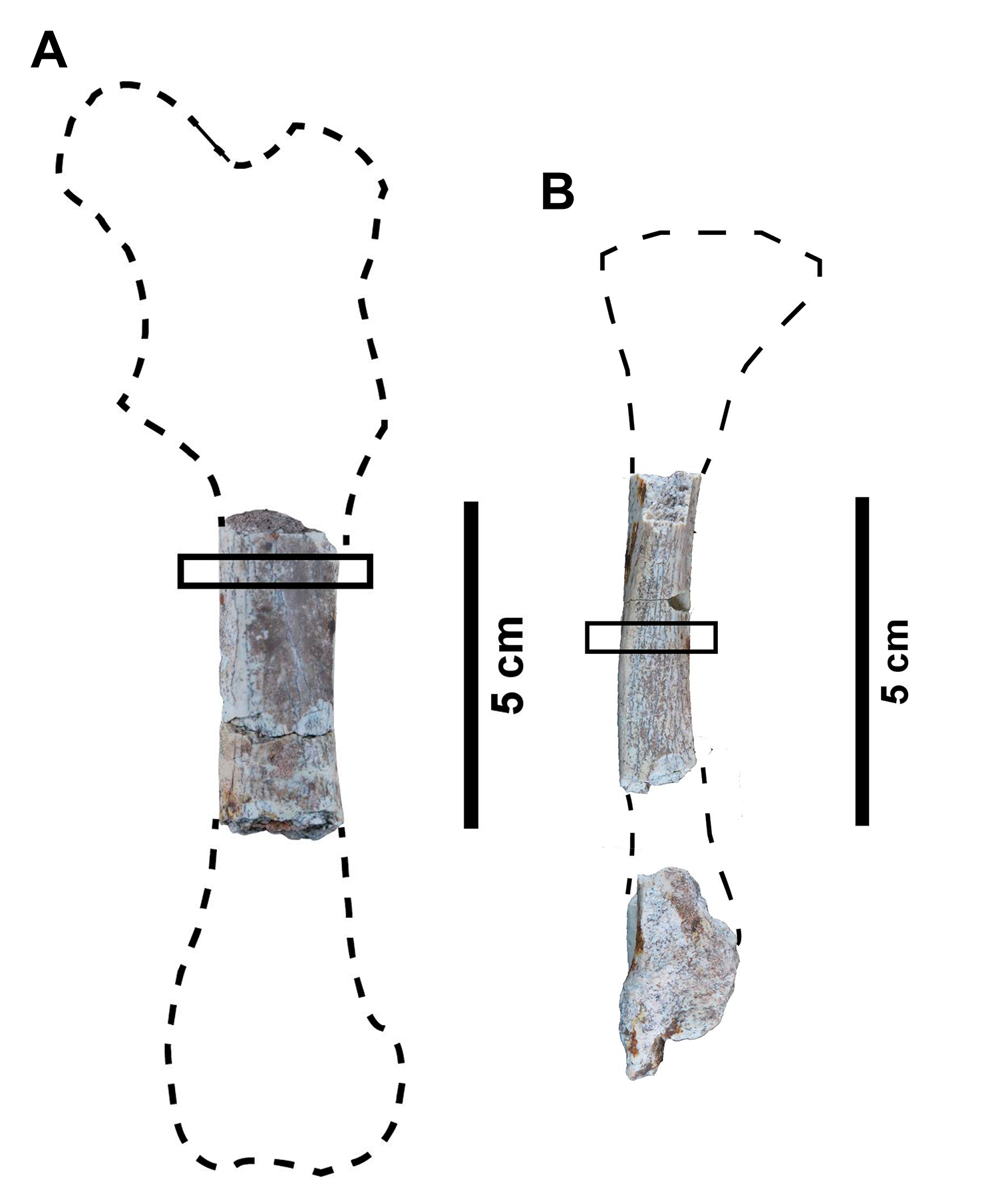

Supplement: S5 Fig — A: Left femur anterior view (GEOUACH.HS.FI.2); B: Left Tibia anterior view (GEOUACH.HS.TI.2). Histological section area is indicated with a rectangle in black. (TIFF) [file pone.0273127.s005.tiff]

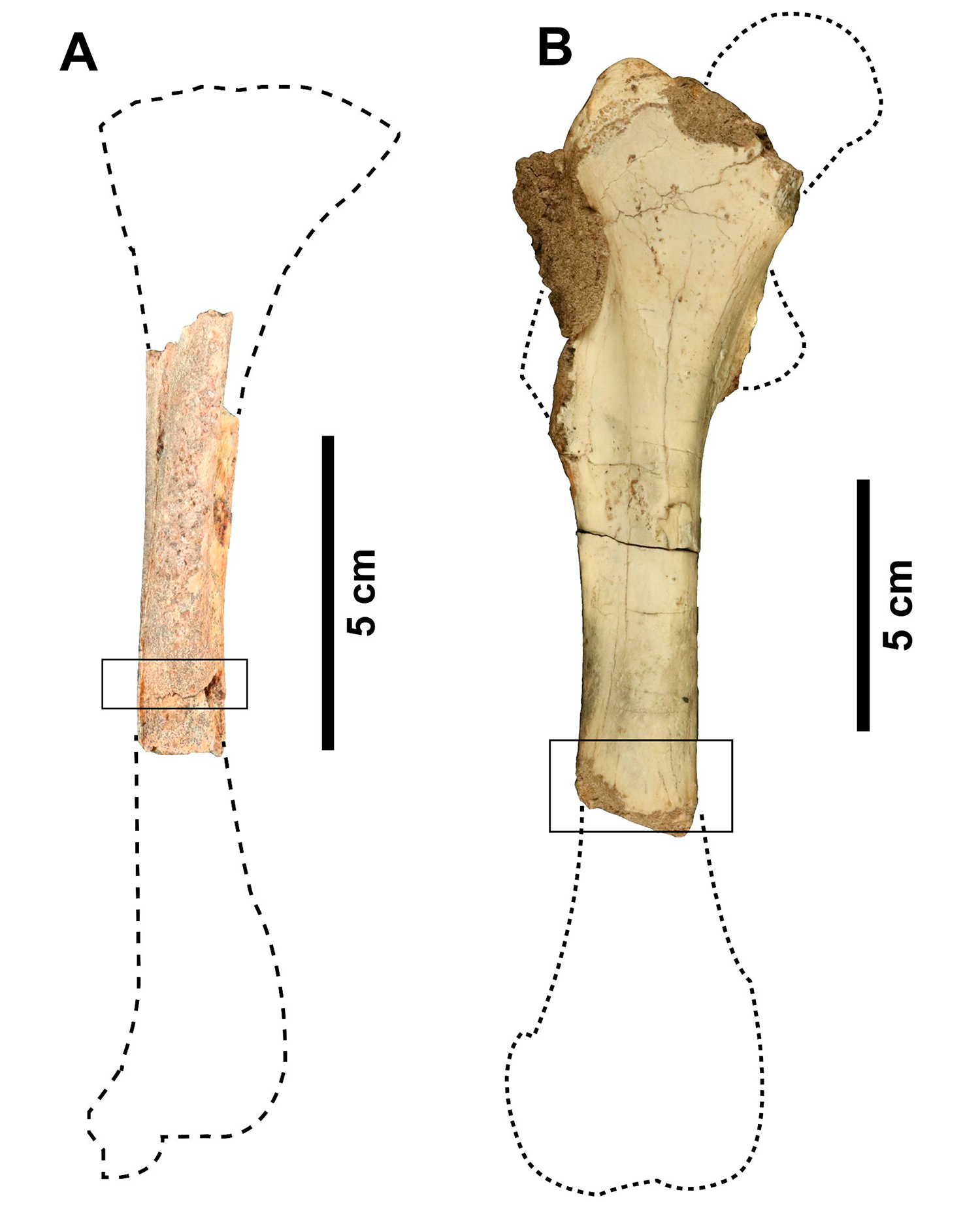

Supplement: S6 Fig — A: Bone element of individual 6 (HUAY15-027). Left tibia anterior view (GEOUACH.HS.TI.3); B: Bone element of individual 7 (HUAY17-02), Right femur anterior view (GEOUACH.HS.FD.1). Histological section area is indicated with a rectangle in black. (TIFF) [file pone.0273127.s006.tiff]
